# Electrolyte formulation strategies for potassium‐based batteries

**DOI:** 10.1002/EXP.20210239

**Published:** 2022-02-15

**Authors:** Ling Ni, Gaojie Xu, Chuanchuan Li, Guanglei Cui

**Affiliations:** ^1^ Qingdao Industrial Energy Storage Research Institute Qingdao Institute of Bioenergy and Bioprocess Technology, Chinese Academy of Sciences Qingdao China

**Keywords:** fundamentals of organic electrolytes, liquid electrolytes, potassium‐based batteries, solid‐state electrolytes

## Abstract

Potassium (K)‐based batteries are viewed as the most promising alternatives to lithium‐based batteries, owing to their abundant potassium resource, lower redox potentials (−2.97 V vs. SHE), and low cost. Recently, significant achievements on electrode materials have boosted the development of potassium‐based batteries. However, the poor interfacial compatibility between electrode and electrolyte hinders their practical. Hence, rational design of electrolyte/electrode interface by electrolytes is the key to develop K‐based batteries. In this review, the principles for formulating organic electrolytes are comprehensively summarized. Then, recent progress of various liquid organic and solid‐state K^+^ electrolytes for potassium‐ion batteries and beyond are discussed. Finally, we offer the current challenges that need to be addressed for advanced K‐based batteries.

## Graphical abstract

This review mainly focuses on the recent progress of various liquid organic and solid‐state K^+^ electrolytes for potassium‐based batteries. First, the key design principles and applications of organic liquid electrolyte in various battery systems are discussed. Then the research status of ionic liquid, gel/polymer, and solid electrolyte are summarized and evaluated. Finally, this review discusses the challenge of various electrolyte/electrode interfaces and puts forward promising research direction and opportunities in the future.

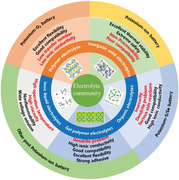

## 1 INTRODUCTION

The clean and sustainable energies could relieve the environmental pollution and fossil fuel crisis. However, these generated energies are intermittent depending on the geographic location and weather conditions. Therefore, it is necessary to seek a large‐scale energy storage systems (ESSs) for efficient storing and outputting the variable energy (such as, wind, tidal, and solar energy). Rechargeable batteries are the most efficient ESS to store and convert chemical energy into electrical energy. Lithium‐based batteries, as one typical rechargeable battery, have dominated the markets of portable electronic devices, and electric vehicles in the past few decades.^[^ ^1^ ^]^ Nevertheless, the scarce reserves and uneven distribution of lithium together with the increasing cost of cobalt also raise the concerns over sustainable supply, simultaneously, accelerate the research of alternative rechargeable battery. Potassium (K)‐base batteries and sodium (Na)‐based batteries provide an alternative solution, owing to abundant reserves of K and Na (2.3 and 1.5 wt%, respectively).^[^ ^2^ ^]^ Moreover, the price of potassium and sodium carbonate (K_2_CO_3_, Na_2_CO_3_) are considerably less expensive than Li_2_CO_3_. Since both K and Na do not alloy with aluminum, the aluminum foil is employed as the anodic current collector, replacing the costly and dense copper in lithium‐based batteries.^[^ ^3^ ^]^

However, potassium‐based batteries potentially offer numerous advantages over sodium‐based batteries. First, K‐based batteries are expected to provide a higher work voltage than sodium‐ion battery (SIB). The lower redox potential of K/K^+^ electrode (−2.93 V vs. *E*_0_) than that of Na (−2.71 V vs. *E*_0_), which guarantee high‐energy density. In addition, it has been confirmed the voltage of the K/K^+^ in propylene carbonate (PC) is lower than Li/Li^+^.^[^ ^4^ ^]^ Then, K‐based batteries could realize high‐rate performance due to the high diffusion coefficient of K^+^ ions. Although potassium has the large atomic radius (1.38 Å), K^+^ has the smallest Stokes’ radius (3.6 Å) in propylene carbonate (PC) solvents owing to weak coulombic interactions of K^+^.^[^ ^5^ ^]^ The first‐principles molecular dynamics simulations also have proved that the diffusion coefficient of K^+^ in EC is three times than that of Li^+^.^[^ ^6^ ^]^ In addition, it also revealed that K^+^ has the lowest solvation energy of (397.5 kJ mol^−1^) in ethylene carbonate (EC), which favor a higher rate capability compared to Li^+^ and Na^+^.^[^ ^7^ ^]^ Furthermore, K^+^ could reversibly intercalate/deintercalate into or from graphite, which is the most used anode material for lithium‐ion batteries.^[^ ^8^ ^]^ On the basis of the advantages mentioned above, K‐based batteries are most possible alternatives to lithium‐ions batteries (LIBs).

Recently, considerable progress has been achieved on K‐based batteries. Specifically, these strategies are focused on designing high‐performance cathode and anode materials, electrolyte, interface engineering, and electrode/battery configurations, etc. At present, vast electrode materials have used in K based batteries. For anode materials, carbon‐based materials,^[^ ^9^ ^]^ alloy negative,^[^ ^10^ ^]^ transition metal oxides/chalcogenides and K or Na‐K alloy anode^[^ ^11^ ^]^ have intensively studied. Among the anode candidates, K metal has higher work voltage and specific capacity (≈687 mAh g^−1^). Moreover, K metal anodes can enable the application of potassium‐free cathodes battery systems, such as, potassium metal batteries (KMBs), potassium superoxide (K‐O_2_), and potassium‐sulfur batteries (K‐S). Cathode materials for K‐ion batteries involve four categories: Prussian blue (PB) and its analogues,^[^ ^12^ ^]^ layered transition metal oxides,^[^ ^13^ ^]^ organic redox‐active molecules, and polyanionic compounds.^[^ ^14^ ^]^ However, high‐compatible electrolyte and optimization of interfaces are still in its infancy.

**FIGURE 1:**
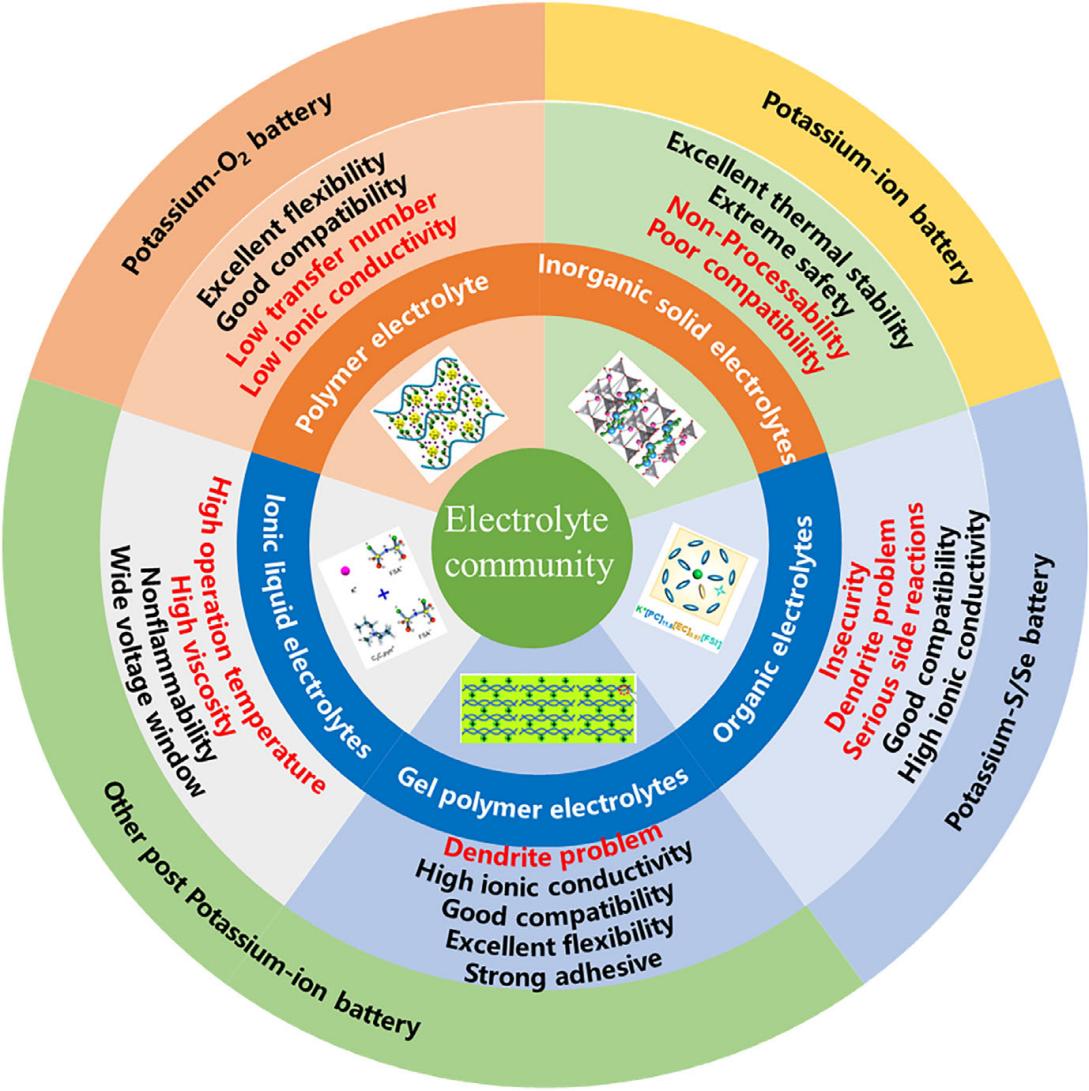
Classifications and characteristics of K‐based batteries

In battery systems, an electrolyte should possess high ionic conductivity, compatible interface, high chemical stability, large electrochemical stability window, ability to inhibit dendrites, and high electronic resistance.^[^ ^15^ ^]^ Up to now, the reported electrolytes for K‐based batteries can be divided into liquid and solid‐state electrolyte (Figure 1A). As for liquid organic electrolyte, the most mature one for K‐based batteries, the compositions are continuously optimized to cater new battery electrodes. For instant, the KClO_4_/PC solutions was first explored for the potassium‐ion batteries (PIBs) applications.^[^ ^16^ ^]^ Recently, KPF_6_ and KFSI salts are also widely investigated in PIBs. However, one of the main concerns of such organic electrolyte is narrow electrochemical stability window, leading to some undesirable side reactions at electrode/electrolyte interfaces along with the formation of solid electrolyte interphase (SEI).^[^ ^17^ ^]^ The ideal SEI could prevent further side‐reactions, and promoting uniform K^+^ diffusion, which is a recognized prerequisite for a metal or insertion/conversion anode. However, there are still several major problems for organic electrolytes, such as, lack of suitable salts and additives, unstable SEI, serious side reactions, high flammability, and K dendrites growth. To address the problems, various electrolytes such as, inorganic electrolytes, ionic liquid (IL) electrolyte, gel polymer, and polymer electrolytes are proposed. Nevertheless, these novel electrolytes suffer from low ionic conductivity at room temperature, and their electrochemical performance for K‐based batteries are almost blank. In brief, the booming development of K‐based batteries will depend on the exploration of matching electrolytes, as well as, in‐depth understanding of interface.

In this review, we begin with common formulation and design principle of K^+^ electrolytes in Section 2, understanding how K^+^ exists and transports across cells, how the interphases are formed and structured at both electrode surfaces. In Sections 3 and 4, we focus on electrolyte design strategies and research progress for potassium‐based batteries, including PIBs, potassium‐metal batteries, K‐S/Se, and K‐O_2_ batteries, from liquid and solid‐state electrolytes. Considering K metal as the counter/reference/work electrode K based batteries, the issue of dendrites growth and unstable K/electrolyte interface are critical needed. Finally, future perspectives and directions are put forward for follow‐up researches.

## 2 ELECTROLYTES FOR K‐BASED BATTERIES

### 2.1 Classifications of electrolytes

Currently, there are two main types of K^+^ based electrolytes electrolyte, liquid electrolytes, and solid‐state electrolytes. Liquid electrolytes involve organic system, aqueous system, gel polymer, and ionic liquid system. Solid‐state electrolytes contain inorganic solid electrolyte (ISE) and polymer system. In this review, only organic, polymer gel, ionic liquid, polymer, or inorganic electrolytes are covered, leaving out aqueous electrolytes.

As observed, organic electrolytes are the most widely used electrolytes in Li/Na/K‐based batteries, owing to their unsurmountable advantages, such as, high ionic conductivity, good compatibility with electrodes.^[^ ^18^ ^]^ Most organic potassium electrolytes are based on solutions of one or more potassium salts in mixed solvents, with a small fraction of functional additives. The solvents have two sides. On the one hand, organic solvents with high dielectric constants and low viscosity enable faster ion mobility and better interfacial wetness. On the other hand, the active reaction, the high volatilization and flammability of organic solvents pose a major safety hazard. Thus, the major challenge of organic electrolytes design is the option of solvents, additives and appropriate concentration. Accordingly, it is of great significance to reduce the amount of solvent for batteries. Gel polymer electrolytes (GPEs) could be considered as the special variation of liquid‐type electrolytes, in which only a small fraction of polymer is employed as the mechanical host, and essentially swollen by liquid electrolytes.^[^ ^19^ ^]^ Although they show improved properties in ionic conductivity and compatibility, the growth of dendrites still remained the serious safety concerns. Ionic liquid (IL) electrolytes exist as liquid state, and consist of organic anions and cations, coupled with soluble K salt.^[^ ^20^ ^]^ The opposite ions are achieved by the thermal melting rather than solvation by solvent. They are demonstrated to possess excellent electrochemical and thermal stability, low volatility, and fire resistance. However, IL electrolytes have lower K^+^ conductivity at room temperature (RT) because of higher viscosity and charge transfer numbers.

Different from organic electrolytes, solid‐state electrolytes exhibit high safety, thermal stability, and mechanical strength, which make they are potential frontier with great potential in academia and industry.^[^ ^15^ ^]^ However, Integration of highly ionic conductive solid electrolytes (SSEs) in solid state batteries remains a challenge, mainly due to the high impedance of the electrolyte/electrode interface. Moreover, at present, both polymer and inorganic electrolytes show low K^+^ conductivity, and electrochemical performance in K‐based batteries is scarce.

### 2.2 The fundamental properties of organic electrolytes

In battery systems, electrolytes work an important role in the performance of batteries. The rate capability, cycle life, coulombic efficiency (CE) of batteries are closely related to the ion transport, solvation effect of K^+^, and electrochemical stability of electrolytes. In this section, we describe fundamental properties (ionic conductivity, ionic transference number, electrochemical stability), how K^+^ exists and moves in bulk electrolyte, how the interphases are formed and structured at both electrode surfaces, and how improve the electrochemical stability window of electrolytes.

#### 2.2.1 Ion transport

**FIGURE 2:**
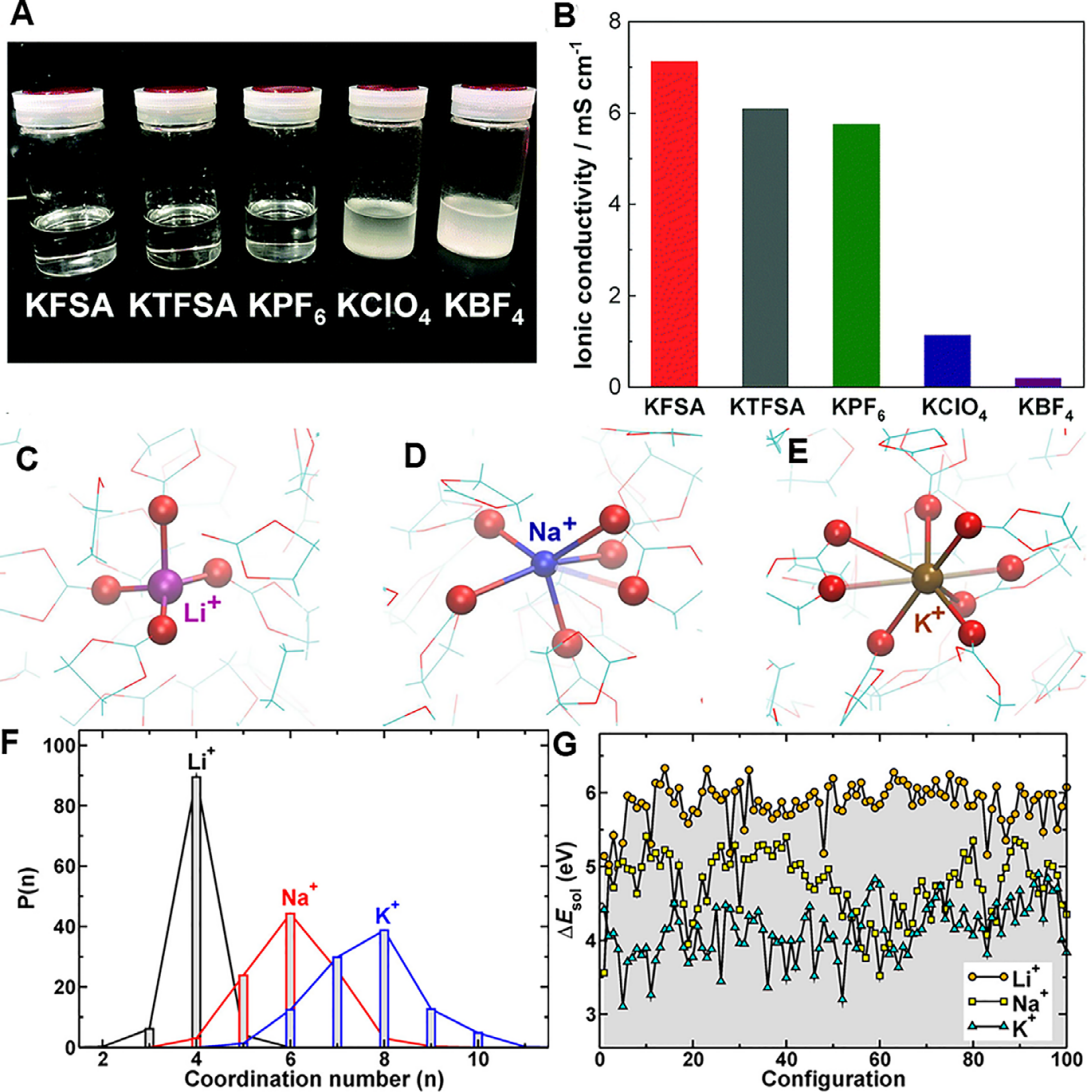
(A) Photograph of mixtures of 0.5 M electrolytes with KFSI, KTFSI, KPF_6_, KBF_4_, and KClO_4_ in PC. (B) Ionic conductivities of the prepared electrolytes at 25°C. Reproduced with permission.^[^ ^23^ ^]^ Copyright 2018, RSC. The solvation structures of (C) Li^+^, (D) Na^+^, and (E) K^+^ in EC. (F) The oxygen coordination number in the first solvation shell of Li^+^, Na^+^, and K^+^ ions. (G) Comparison of solvation energies (Δ*E*_sol_) of Li^+^, Na^+^, and K^+^ ions. Reproduced with permission.^[^ ^6^ ^]^ Copyright 2017, ACS

Ionic conductivity is a fundamental measure for electrolyte, which quantifies how fast ions move between cathode and anode, and in part determines the power output of a cell. The ionic conductivity of electrolyte is relevant to the dielectric properties and viscosity of the solvent, the interaction between solvent and cations, solubility of the salt, and concentration. As well known, solvent with high dielectric constant (ε, to dissolve the salt) and low viscosity (*η*, to facilitate ion transport) exhibits high ionic conductivity for their solutions. However, these two parameters are contradictory. Thus, mixed solvents of either high dielectric permittivity or low viscosity are needed. The cyclic carbonates like propylene carbonate (PC) or ethylene carbonate (EC) and linear carbonates, such as, dimethyl carbonate (DMC), diethyl carbonate (DEC), and ethyl methyl carbonate (EMC), to date, are the most promising electrolytes for alkalis battery applications. So far, in such mixed solvents, optimize ion conductivity reach as high as 5–10 mS cm^−1^.^[^ ^21^ ^]^ Okoshi et al. evaluated 27 organic solvents, in terms of geometric structure, desolvation energy, and interaction energy of K^+^, and concluded that the interaction between K^+^ and solvents played a dominant role in mobility.^[^ ^22^ ^]^ The solubility of salt is related with its lattice energy, and low dissociation energy promotes the dissolution of K salt. The effects of common K salts on transport properties of electrolyte have been explored by Hosaka et al.^[^ ^23^ ^]^ Figure 2A,B demonstrates the solubility and ionic conductivity of 0.5 M KPF_6_, KFSI, KTFSI, KClO_4_, and KBF_4_ in PC. The KPF_6_, KFSI, KTFSI completely dissolved, while vast salt powder visually remained in the KClO_4_ and KBF_4_ mixtures. The 0.5 M KFSI, KTFSI, and KPF_6_ in PC solutions showed high ionic conductivities in the scope of 5–7 mS cm^−1^, while KClO_4_ and KBF_4_ exhibited lower. Generally, K salts with large size of anions have a high solubility, such as, KFSI and KTFSI.^[^ ^24^ ^]^ Thus, KFSI based electrolytes displayed higher conductivity than that of KPF_6_ solutions in various solvents, for instance, EC/DEC, PC, and dimethyl ether (DME).^[^ ^25^ ^]^ Electrolyte concentration is also a key strategy for regulating ionic conductivity. In the past for a long time, 1 M is considered as the most optimal concentration at which the ion conductivity is maximal.^[^ ^26^ ^]^

Transference number is another transport property, which quantifies the valid fraction of ion conductivity. The fraction of the current carried by cation is for ongoing electrochemical reactions, thus, high ionic transference number of cation (*t*_+_) is beneficial for improving rate performance.^[^ ^15^ ^]^ It had proved that even modest improvements in *t*_+_, for example, to *t*_+_ ≈ 0.7, would be beneficial to quickly charge the battery.^[^ ^27^ ^]^ However, the ionic conductivity obtained by electrochemical impedance spectroscopy is the apparent conductivity, which involves the contribution of both cations and anions, and few studies accurately measure the true transference number.^[^ ^28^ ^]^ Compared to Li^+^ (*t*_Li+_, 0.39), K^+^ possesses higher transference number (0.43) in 1 M KFSI in EC/DEC = 1:1, due to low charge density of K^+^.^[^ ^29^ ^]^ In binary molten electrolyte LiCl/KCl system, the mobility of K^+^ was greater than that of the smaller Li^+^ cation, too. A high K^+^ transference number is beneficial for reducing concentration polarization within batteries. From a broader perspective, although high ion conductivity and transference number does not suffice to fully characterize an electrolyte and predict its behavior in a K ion cell, these properties are prerequisites.

#### 2.2.2 K^+^‐solvation

Cations are always solvated by solvent molecules in electrolytes through ion‐solvent interaction, which is defined as M^+^‐solvation. The M^+^‐solvation effect is correlated with the ionic kinetics, SEI components, and the stability of batteries.^[^ ^30^ ^]^ The behaviors of active M^+^ on interfaces are always accompanied by the solvation/desolvation processes, which have great influence on the M^+^ migration. Figure 2C–E and Table 1 schematically show the solvation structures and energy of Li^+^, Na^+^, K^+^ in common organic solvents. In contrast to Li^+^, which exhibited a well‐defined tetrahedral arrangement with EC molecules, the larger Na^+^ and K^+^ showed more disordered and flexible solvation structures. Specifically, Na^+^ and K^+^ exhibited increased average coordination numbers of 5.7 and 7.6 (Figure 2F), which were mostly composed of carbonyl oxygen and a small fraction of ether oxygen. Solvation structure and energy have a joint effect on the electrochemical properties of K^+^ (Figure 2G). Typically, the larger solvation structure may prevent K^+^ intercalation/deintercalation possess, thus lead to slow kinetics. While the smaller desolvation energy was beneficial for solvation/desolvation processes in K^+^ electrolyte.^[^ ^31^ ^]^ Furthermore, the solvation sheath molecules are considered to transfer with K^+^, thus, the solvent molecules reduced by electrons and constitute the main ingredient of SEI.^[^ ^32^ ^]^ Therefore, the option of electrolyte components plays a pivotal role on SEI.

**TABLE 1:** Desolvation energy of Li^+^, Na^+^, and K^+^ in typical organic solvents

	Desolvation energy (kJ mol^−1^)							
Ion	DEC	PC	EMC	FEC	VC	TMP	BC	DMF
Li^+^	205.6	215.8	199.1	188.8	191.4	249.1	219.5	230.1
Na^+^	147.9	158.2	143.1	136.2	138.3	181.2	161.4	165.5
K^+^	105.1	119.2	101.6	100.5	102.2	135.4	121.9	122.8

*Note*: FEC, Fluoroethylene carbonate; TMP, Trimethyl phosphate; VC, Vinylene carbonate; BC, Butylene carbonate; DMF, Dimethyl formamide.

*Source*: Reproduced with permission.^[7a]^ Copyright 2017, IOP.

#### 2.2.3 Electrochemical stability

**FIGURE 3:**
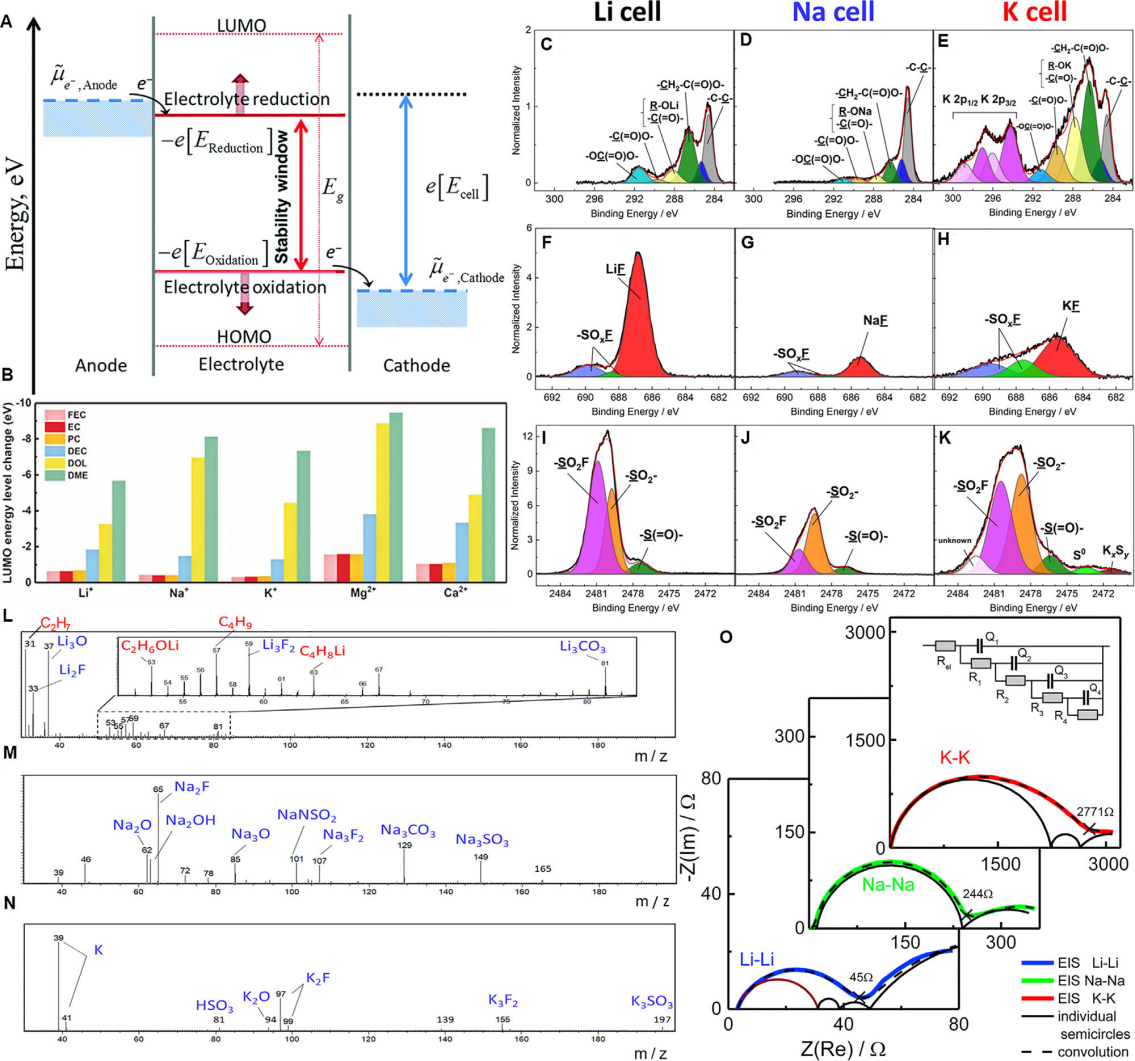
(A) The anode and cathode potential range of electrolyte. Reproduced with permission.^[^ ^33^ ^]^ Copyright 2008, RSC. (B) Comparison of LUMO energy level changes between the ion‐solvent complexes and pure solvents. Reproduced with permission.^[35a]^ Copyright 2018, Wiley. (C–E) C 1s, (F–H) F 1s, and (I–K) S 1s HAXPES spectra of the hard carbon electrodes in (C, F, I) Li, (D, G, J) Na, and (E, H, K) K cells with 1 M AFSI/PC (A = Li, Na, and K) electrolyte. TOF‐SIMS positive ion spectra of the hard carbon electrodes after 10 cycles in (L) Li, (M) Na, and (N) K cells. Reproduced with permission.^[^ ^39^ ^]^ Copyright 2020, ACS. (O) Electrochemical impedance spectroscopy of symmetrical alkali‐metal cells. Reproduced with permission.^[^ ^42^ ^]^ Copyright 2017, Elsevier

Meanwhile, the solvation structure has a key impact on the chemical stability of electrolytes. Figure 3A shows the electron transfer and energy state on electrode/electrolyte interface.^[^ ^33^ ^]^ The frontier molecular orbital theory predicts the reductive/oxidative stability of various electrolytes by calculating corresponding lowest unoccupied molecular orbital (LUMO)/highest occupied molecular orbital energy, respectively.^[^ ^34^ ^]^ When solvents or anions coordinate with cations, the coordinated cations attract electrons from solvent molecules and reduce the LUMO energy level of electrolytes. When the reductive decomposition potential of anode below the LUMO, some side reactions would occur at interfaces, for instance electrolyte reduction and gas evolution, as well as, formation of the passivation layer (SEI).^[^ ^35^ ^]^ This principle is applicable to many other divalent cation‐solvent systems, including Mg^2+^, Zn^2+^, and Ca^2+^ cations and ester and ether solvents (Figure 3B).^[^ ^36^ ^]^ The SEI has an important impact on the potassium‐based batteries performance, including the initial coulombic efficiency, hysteresis voltage, cycle life, and integrated safety.^[^ ^37^ ^]^ However, it seems to be more difficult to form sufficient passivation layers on K metal anode in comparison to Li metal, including stability, mechanical, and transfer properties.

#### 2.2.4 Solid electrolyte interphase formation

It is widely accepted that the SEI properties, including its uniformity, solubility, and ionic conductivity, account for a substantial part of anode electrochemical performances. SEIs are required in organic alkalis‐based batteries, which work at below thermodynamically stable potential of electrolytes. In 2001, Aurbach et al. first reported the surface films on the gold electrodes in PC solutions with Li/K perchlorate salts were relative more stable than those in NaClO_4_ solution.^[^ ^16^ ^]^ It was concluded that Lewis acidity, and size of cation, as well as, the degree of polymerization were crucial factors to achieve strong coulombic interaction or bond strength of film species.^[^ ^38^ ^]^ Recently, Komaba et al. conducted comparative experiments in SEI on hard carbon electrodes via hard X‐ray photoelectron spectroscopy (HAXPES) and time‐of‐flight secondary ion mass spectrometry (TOF‐SIMS).^[^ ^39^ ^]^ Figure 3C–E shows the normalized C 1s HAXPES spectra of hard carbon electrodes cycled 10 times in Li, Na, and K cells, respectively. All these peaks attributed to the R─OK, ─CH_2_─COO─, ─OC( = O)O─, and ─C( = O)─ bonds in alkoxides and alkyl carbonates, which were the reduction products of the PC. Obviously, Li and K cell displayed higher C 1s peak intensities than that of Na cell, indicating thicker SEI in the Li/K cell with large amounts of organic/inorganic components. Carefully identify, the SEI in the K cell had a different amount of inorganic species derived from FSI^−^ from that in the Li cell. The F 1s and S 1s spectra (Figure 3F–K) in the K cell showed the highest amounts of ─SO_2_F, > S = O, and ─SO_2_─, but with lower content of KF than LiF. Moreover, the peaks attributed to elemental S and K*_x_* S*_y_* were only found in the K cell, suggesting a quite different SEI formation mechanism of anions.^[^ ^40^ ^]^ The electrode surfaces at a depth of ≈1 nm was conducted by TOF‐SIMS (Figure 3L–N). In the Li cell, both organic fragments (e.g., C_2_H_6_OLiC and _2_H_7_) and inorganic (e.g., Li_2_F and Li_3_O) were observed. Nevertheless, mainly inorganic (e.g., K_2_O, Na_2_O, NaF, and KF) were observed in the TOF‐SIMS spectra, which indicated a comparatively high solubility of organic compounds in K/Na‐ion SEI.^[^ ^38^, ^41^ ^]^ The above results revealed that the optimal formulation of electrolytes for K‐ion batteries should not be same with those used for Li and Na. Hess also reported the Li, Na, K metal anode SEI possessed increased ohmic resistor (Figure 3O).^[^ ^42^ ^]^ Numerous experimental and computational results strongly indicate that the deep understanding formation mechanism, composition structure, stability, and influencing factors of SEI membrane and finding an effective way to improve the performance is a breakthrough in realizing high‐performance K‐based batteries.

### 2.3 Organic electrolyte components

#### 2.3.1 Solvents

The organic electrolytes are composed of three major components solvents, K salts, and additives. Solvent is the most important component of electrolyte. An ideal solvent should meet the diverse requirements, for example, high dielectric constants, low viscosity, low melting point (*T*_m_), high boiling point (*T*_b_), high flash point (*T*_f_), high stability, environmentally‐friendly, and low‐cost. Therefore, solvents of very different physical and chemical natures are often mixed to perform various functions simultaneously. The most commonly used solvents fall into three families: Carbonate ester, ether, and phosphate ester.

Carbonate ester is the main part of solvents in PIBs due to high electrochemical stability. The commonly used are propylene carbonate (PC), ethylene carbonate (EC), EMC, DEC, and DMC. Ether solvents have good compatibility with alkali metals, while the poor oxidation resistance limits their applications. The conventional ether solvents include DME, diethylene glycoldimethyl ether (DEGDME), triethylene glycol dimethyl ether (TEGDME), and 1,3‐dioxacyclopentane. With the effort to developing nonflammable electrolytes, organic phosphate as fire‐retardant solvents have been reported,^[^ ^57^ ^]^ such as, triethyl phosphate (TEP), and trimethyl phosphate (TMP) (Table 2).

**TABLE 2:** Specific solvents and salts for potassium‐ion batteries reported in 2021

Electrode	Electrolyte	Capacity retention (mAh g^−1^)	Voltage window (V)	Ref.
KTiOPO_4_	0.8 M KPF_6_ in EC/DEC = 1	78.4% after 10,000 cycles (20 C)	0–3	^[^ ^43^ ^]^
Hollow hierarchical porous olive‐like carbon	0.8 M KPF_6_ in EC/DEC = 1	>90% after 800 cycles (1 A g^−1^)	0.01–3	^[^ ^44^ ^]^
F, N co‐doped carbon nanosheets	0.8 M KPF_6_ in EC/DEC = 1	69% after 5000 cycles (2 A g^−1^)	0.01–3	^[^ ^45^ ^]^
TiO_2_ ww/CN	1 M KFSI in EC/DMC = 1	75.6% after 6000 cycles (5 A g^−1^)	0.01–3	^[^ ^46^ ^]^
Fe*_x_*_−1_Se*_x_*/MXene/FCR	0.8 M KPF_6_ in EC/DMC = 1	67.7% after 2000 cycles (10A g^−1^)	0.01–3	^[^ ^47^ ^]^
Ultrasmall CoP nanoparticles	0.8 M KPF_6_ in EC/DEC = 1	74.3% after 2800 cycles (100 mA g^−1^)	0.01–3	^[^ ^48^ ^]^
Antimony (Sb)	4.0 M KFSI in DME	95.4% after 200 cycles (200 mA g^−1^)	0.01–2	^[^ ^49^ ^]^
BiPS_4_‐CNT hybrid	1 M KFSI in EC/DEC = 1	72.5% after 600 cycles (1 A g^−1^)	0.01–3	^[^ ^50^ ^]^
Lamellar tetrapotassium pyromellitic	5.0 M KFSI in DME	83 % after 1000 cycles (500 mA g^−1^)	0.01–3	^[^ ^51^ ^]^
K_1/2_Mn_5/6_Mg_1/12_Ni_1/12_O_2_	0.8 M KPF_6_ in EC/PC = 1	70.4% after 200 cycles (120 mA g^−1^)	1.5–3.9	^[^ ^52^ ^]^
K_0.5_V_2_O_5_·0.5H_2_O	KFSI in TEP (weight ratio of 1 : 2)	74% after 400 cycles (100 mA g^−1^)	2.0–4.5	^[^ ^53^ ^]^
K_2_Mn[Fe(CN)_6_	2.5 M in TEP	80% after 7800 cycles (500 mA g^−1^)	2.7–4.4	^[^ ^54^ ^]^
K_1.5_VOPO_4_F_0.5_	0.5 M KPF_6_ in EC/PC = 1	86% after 300 cycles (1 C)	1.8–4.5	^[^ ^55^ ^]^
K_4_[Mn_2_Fe](PO_4_)_2_(P_2_O_7_)	0.5 M KPF_6_ in EC/DEC = 1	83% after 300 cycles (C/3)	2.0–4.3	^[^ ^56^ ^]^

**FIGURE 4:**
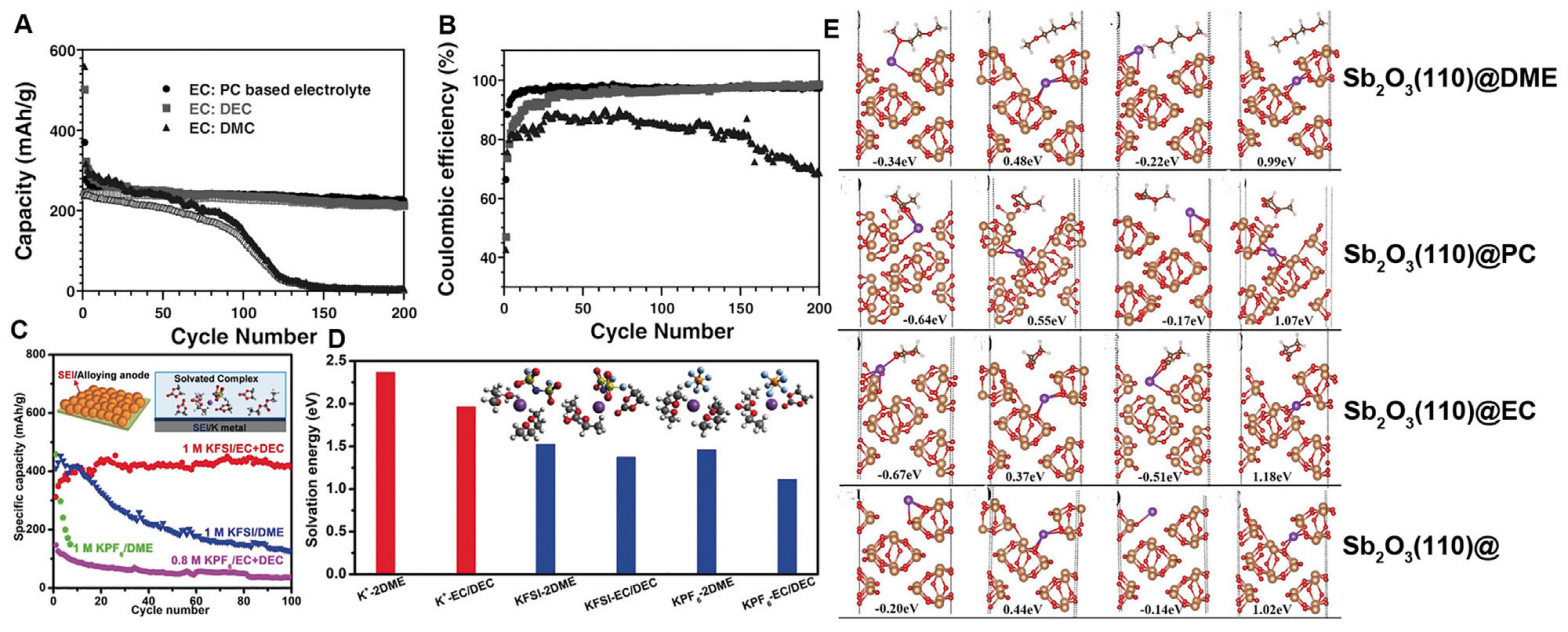
Comparison of (A) cycling performance and (B) coulombic efficiency of graphite anodes in KPF_6_ based EC/PC, EC/DEC, and EC/DMC electrolytes. Reproduced with permission.^[^ ^58^ ^]^ Copyright 2016, Wiley. (C) Cycling performance curves of RP/C anodes in different electrolytes at current density of 50 mA g^−1^ (D) Solvation energies of solvated K^+^‐solvent complexes. Reproduced with permission.^[^ ^60^ ^]^ Copyright 2019, Wiley. (E) The possible adsorption sites for K^+^ in surfaces of (110)@DME, (110)@PC, (110)@EC, and (110)@, respectively. Reproduced with permission.^[^ ^61^ ^]^ Copyright 2020, Wiley

The effect of solvents on anode electrode performance has been widely explored. EC become indispensable ester solvent for a number of electrolyte properties. Meanwhile, the EC/PC‐based electrolytes show more stable and compatible with graphite at low voltage. Wang et al. investigated the effect of three mixed ester solvents: EC/PC, EC/DMC, and EC/DEC on the electrochemical performance of graphite in K‐ion batteries.^[^ ^58^ ^]^ As shown in Figure 4A,B, the 1 M KPF_6_ in EC/PC electrolyte showed high coulombic efficiency and long cycle life. The authors inferred that the worse performance was related with the poor stability of DEC and DMC at low voltage, which also confirmed by Ponrouch and Kang.^[^ ^59^ ^]^ Moreover, new battery electrodes would incur new electrolyte compositions. Guo et al. compared the electrochemical performance of red phosphorus (RP/C) alloy anode in EC/DEC and DME solvent, and demonstrated EC/DEC based electrolyte stabilized both K metal and RP electrodes for highly stable PIBs (Figure 4C).^[^ ^60^ ^]^ Furthermore, density functional theory (DFT) calculations indicated that moderate solvation energy of KFSI based EC/DEC complex could facilitate K^+^ ion diffusion and desolvation on RP/C anode surface, and alleviate side reactions of electrolyte (Figure 4D). While Mai's group confirmed Sb_2_O_3_‐RGO composite using DME electrolyte exhibited lower energy exchange and migration barrier than other PC, or EC for K ions (Figure 4E).^[^ ^61^ ^]^ Except for the traditional ester/ether‐based electrolyte mentioned above, the new nonflammable phosphate ester electrolytes have been explored. For example, Guo et al. developed a new moderately concentrated KFSI/TEP electrolyte, in which K metal achieved a high average coulombic efficiency of 99.6% and small plating/stripping overpotential.^[^ ^62^ ^]^

#### 2.3.2 Salts

An ideal salt for ambient rechargeable potassium batteries should meet the following minimal requirements, such as, high solubility, good electrochemical/chemical/thermal stability, low‐cost, and non‐toxic. Unfortunately, the kinds of K salts reported so far are rare. The common salts in K‐based batteries include KBF_4_, KClO_4_, KPF_6_, KN(SO_2_F)_2_ (KFSI), KN(CF_3_SO_2_)_2_ (KTFSI), and KCF_3_SO_3_,^[^ ^39^ ^]^ and more research is focus on the applications of KPF_6_, and KFSI.

For KBF_4_ and KClO_4_ salts, the poor solubility (0.05 M) in typical aprotic solvents lead to the low conductivity of the electrolyte, limiting their potential in potassium based batteries.^[^ ^63^ ^]^ In addition, KClO_4_ is rarely applied due to its strong oxidation. The high thermal stability and the passivation to Al foil make KPF_6_ based electrolyte be extensively studied. The major objective of KPF_6_ based electrolyte is adjusting functional solvents and additives, due to its moderate concentration (0.5–1.5 M). Lu et al. significantly improved the cycling stability of graphite anodes applied a high‐temperature precycling step to regulate the solvation structure of K^+^‐solvents in KPF_6_ based electrolyte.^[^ ^64^ ^]^

However, KPF_6_ is extremely sensitive to oxygen and water, and easy to be decomposed HF, PF_5_, and POF_3_. Imides (KFSI, KTFSI) as new‐type potassium salts, are considered as the most advantageous for negative electrodes.^[^ ^65^ ^]^ Specifically, Komaba et al. demonstrated the 3.9 M KFSI/DME realized the smallest polarization of 25 mV.^[^ ^66^ ^]^ KFSI/DME electrolyte could significantly improve the electrochemical performance of such kinds of electrodes, that is, carbonaceous material, alloy, and sulfide anodes by building highly stable SEI.^[^ ^67^ ^]^ However, low concentration KFSI based electrolytes exhibit Al corrosion at potential of ≈4.0 V versus K/K^+^.^[^ ^68^ ^]^ Therefore, there is a long way to design optimal electrolyte for potassium based batteries.

#### 2.3.3 Electrolyte additives

In general, additive is a kind of component with a mass fraction below 5%, but it is an indispensable part of the electrolytes in improving the target characteristics, including SEI forming agent, cathode protection agent, and safety protection agent, and so on.^[^ ^69^ ^]^ The typical roles of additives have already been confirmed in LIBs and SIBs. Therefore, additive is an important hotspot for high‐performance potassium based batteries.

Currently, there are no specific potassium ion electrolyte additives. Fuoroethylene carbonate (FEC) can improve the stability of solvent under high voltage, and at the same time, FEC can effectively improve the interface stability of hard carbon,^[^ ^70^ ^]^ alloy anode with large volume expansion (such as, Si),^[^ ^71^ ^]^ and high‐voltage cathode^[^ ^72^ ^]^ in LIBs and SIBs. Thus, the influence of FEC on cathode and anode in PIBs has also been studied. In 2017, the different effect of FEC on Prussian blue analogue (PBA) cathode in K half cells was investigated by Komaba et al.^[^ ^73^ ^]^ The 2 vol% FEC additive based electrolyte could improve the initial coulombic efficiency of K‐MnHCFe (from 61% to 90%), but the capacity retention was still low. Nazar et al. reported the similar conclusion that FEC enhanced the coulombic efficiency because it could not completely prevent electrolyte side reactions with successively consuming upon cycling.^[^ ^74^ ^]^ Moreover, FEC additives play a negative role in most anode electrodes. In K/Sn_3_P_4_ half cells, FEC accelerated the decomposition of the electrolyte, leading to greatly increased the polarization during cycling.^[^ ^75^ ^]^ Similar electrochemical behavior also exists in graphite and GeP_5_ anode.^[^ ^73^, ^76^ ^]^

Apart from FEC, Komaba et al. systematically concluded the negative influence of well‐known additives on K metal plating, including fluoroethylenecarbonate (DFEC), vinylene carbonate (VC), and ethylenesulfite (ES).^[^ ^66^ ^]^ For safer PIBs, Ming et al. first discovered that ethylene sulfate (DTD) was a promising additives, which made the electrolyte of 1.0 M KFSI in TMP more compatible with graphite anode.^[^ ^77^ ^]^ It was concluded that the DTD had a stronger coordination capability compared to that of TMP solvent. Thus, the DTD could replace one proportion of TMP solvent and participate in constructing the first solvation shell of central K^+^ ions, thereby determine the interfacial behaviors of K^+^‐solvent on electrode interface, and affecting the graphite performance. In addition to these solvent‐based additives, small amount of potassium difluorophosphate and blending salts have been developed recently.^[^ ^78^ ^]^

## 3 CURRENT STATE OF LIQUID ELECTROLYTES FOR K‐BASED BATTERIES

### 3.1 Scope of K‐based batteries

**FIGURE 5:**
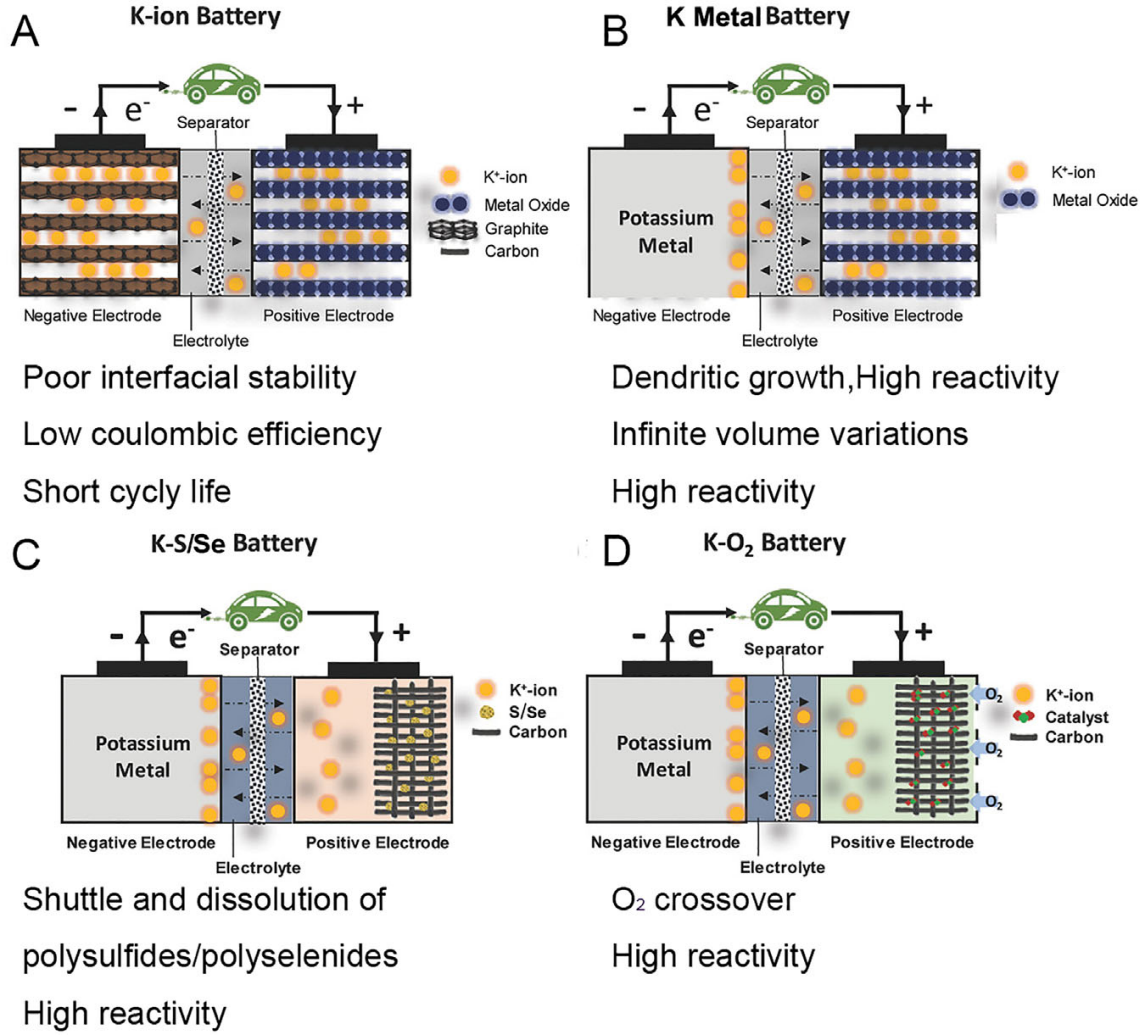
Schematically illustrating the work mechanism and disadvantage of rechargeable potassium‐based batteries: (A) K‐ion, (B) K metal batteries, (C) K‐S/Se, and (D) K‐O_2_. Reproduced with permission.^[^ ^82^ ^]^ Copyright 2018, Wiley

Potassium‐based batteries work in a similar rocking‐chair mechanism that Li^+^‐based batteries do. In PIBs, generally, potassiated transition‐metal compounds work as cathodes, and carbonaceous materials, such as, hard carbon, graphite, as anodes for charge storage (Figure 5A). The research of PIBs much fell behind the Li/Na based batteries due to the larger size of K^+^ and high activity of K metal. Until 2010, Yang et al. discovered the phenomenon of K^+^ intercalation in graphite based on molten KF electrolyte at high temperature.^[^ ^79^ ^]^ Then, in 2013, Wu et al. developed the first potassium‐oxygen batteries (K‐O_2_) (Figure 5D) in 0.5 M KPF_6_/DME electrolyte with low charge–discharge polarization. The development of K‐O_2_ is earlier than the discover of graphite anodes. In 2014, K‐S batteries were discovered with ordered mesoporous carbon (CMK‐3)/sulfur compounds as cathode paired with K metal anode.^[^ ^80^ ^]^ In 2015, the storage performance of graphite in carbonate ester (EC/DEC) electrolyte was demonstrated by Komaba et al. and Ji et al.^[^ ^81^ ^]^ Since then, various alloying, conversion anodes, and Prussian blue (PB), layered metal oxides, polyanionic compounds have been intensively investigated. In addition, there are numerous original works committed to designing advanced electrolytes and optimizing the electrode/electrolyte interface. These instructive works will discuss in the following sections. So far, KMBs (Figure 5A), potassium‐sulfur/selenide (K‐S/Se) (Figure 5C) have emerged as promising candidates for EES due to low costs and high specific energy density. However, during electrochemical processes, intermediate products are soluble and can shuttle across the cells, leading to low columbic efficiency, high self‐discharge rate, and sharp capacity decay (Figure 5).

### 3.2 Liquid electrolytes for potassium‐ion batteries

Electrolytes in batteries must cater to the needs of electrodes, and new battery electrodes would incur new electrolyte compositions. At present, most researches focus on either single cathode or anode materials in PIBs with potassium as reference electrodes. Hence, Tables 3, and 4 show the recent progress of suitable K^+^ electrolytes for cathodes (Prussian blue and analogues, polyanion compounds, layered metal oxides, and organic materials) and anodes (intercalation, conversion, and alloying materials)

**TABLE 3:** Summary of the electrolytes on various cathodes performance in PIBs

Category	Cathode	Electrolyte	Capacity (mAh g^−1^)	Capacity retention	Ref.
Prussian blue analogues	K_2_Ni_0.05_Fe_0.95_Fe(CN)_6_	0.8 M KPF_6_ in EC/DEC (1/1) with 1 wt% FEC	135 at 20 mA g^−1^	83.1%@300 cycles (0.1 A g^−1^)	^[^ ^83^ ^]^
	K_0.220_Fe[Fe(CN)_6_]_0.805_⋅4.01H_2_O	0.8 M KPF_6_ in EC/DEC	65.0 at 100 mA g^−1^	86.5%@150 cycles (0.1 A g^−1^)	^[^ ^84^ ^]^
	K_0.3_Ti_0.75_Fe_0.25_[Fe(CN)_6_]_0.95_⋅2.8H_2_O	0.8 M KPF_6_ in EC/DEC (1/1) with 5 wt % FEC	136.7 at 50 mA g^−1^	64.7%@100 cycles (0.1 A g^−1^)	^[^ ^85^ ^]^
	K_1.89_Mn[Fe(CN)_6_]_0.92_·0.75H_2_O	Saturated KClO_4_ in PC with 10 wt% FEC	146.2 at 0.2 C	141.4%@100 cycles (1 C)	^[12a]^
	K_2_Ni_0.36_Co_0.64_Fe(CN)_6_	0.8 M KPF_6_ in EC/DEC (1/1) with 1 wt% FEC	90 at 20 mA g^−1^	88.2%@300 cycles (0.02 A g^−1^)	^[^ ^86^ ^]^
	Polypyrrole‐coated K‐rich iron hexacyanoferrate	0.8 M KPF_6_ in EC/DEC	88.9 at 50 mA g^−1^	85%@500 cycles (1 A g^−1^)	^[^ ^87^ ^]^
	K_1.69_Mn[Fe(CN)_6_]_0.85_·0.4H_2_O	2.5 M KPF_6_ in diglyme	105 at 0.2 C	68%@30 cycles (0.1 C)	
Layered transition metal oxides	δ‐K_0.51_V_2_O_5_	0.8 M KPF_6_ in PC with 5 wt% FEC	125 at 50 mA g^−1^	61.3%@100 cycles (0.1 A g^−1^)	^[^ ^88^ ^]^
	K_0.3_MnO_2_	1 M KPF_6_ in EC/DMC	136 at 0.1 C	57% @ 685 cycles (0.1 C)	^[^ ^89^ ^]^
	K_0.83_[Ni_0.05_Mn_0.95_]O_2_	0.5 M KPF_6_ in EC/DEC	155 at 52 mA g^−1^	83% @ 200 cycles (0.52 A g^−1^)	^[^ ^90^ ^]^
	K_0.54_[Co_0.5_Mn_0.5_]O_2_	0.5 M KPF_6_ in EC/DEC	125 at 20 mA g^−1^	62%@500 cycles (0.5 A g^−1^)	^[^ ^91^ ^]^
	K_0.44_Ni_0.22_Mn_0.78_O_2_	0.8 M KPF_6_ in EC/DEC (1/1)	125.5 at 10 mA g^−1^	67%@500 cycles (0.2 A g^−1^)	^[^ ^92^ ^]^
	P2‐K*_x_* CoO_2_	1 M KFSI in EC/DEC (1/1)	60 at 11.8 mA g^−1^	91.6%@30 cycles (0.011 A g^−1^)	^[^ ^93^ ^]^
Polyanion compounds	K_3_V_2_(PO_4_)_3_/C	0.8 M KPF_6_ in EC/DEC (1/1)	50 at 20 mA g^−1^	99.1%@100 cycles (0.02 A g^−1^)	^[^ ^94^ ^]^
	K_2.95_Rb_0.05_V_2_(PO_4_)_3_/C	0.8 M KPF_6_ in EC/DMC (1/1)	44 at 20 mA g^−1^	95.4%@100 cycles(0.2 A g^−1^)	^[^ ^95^ ^]^
	K_3_V_2_(PO_4_)_2_F_3_	1 M KPF_6_ in EC/PC (1/1)	83 at 100 mA g^−1^	97%@100 cycles (0.01 A g^−1^)	^[^ ^96^ ^]^
	KVOPO_4_	0.5 M KPF_6_ in EC/FEC (1/1)	115 at 24 mA g^−1^	86.8%@100 cycles (0.06 A g^−1^)	^[^ ^97^ ^]^
	K_4_Fe_3_(PO_4_)_2_(P_2_O_7_)	0.5 M KPF_6_ in EC/PC/FEC (20/20/1)	118 at 6 mA g^−1^	82%@500 cycles (5 C)	^[^ ^98^ ^]^
	KTi_2_(PO_4_)_3_	0.5 M KPF_6_ in EC/DEC (1/1)	126 at 12.8 mA g^−1^	89%@500 cycles (0.1 C)	^[^ ^99^ ^]^
	KFeC_2_O_4_F	1 M KPF_6_ in EC/PC (1/1)	112 at 200 mA g^−1^	94%@2000 cycles (0.2 A g^−1^)	^[^ ^100^ ^]^
Organic materials	PTCDA	0.5 M KPF_6_ in EC/DEC (1/1)	131 at 10 mA g^−1^	76%@2000 cycles (0.05 A g^−1^)	^[^ ^101^ ^]^
	PAQS	0.5 M KTFSI in DOL/DME (1/1)	200 at 20 mA g^−1^	64%@200 cycles (0.2 A g^−1^)	^[^ ^102^ ^]^
	PTPAn	0.8 M KPF_6_ in EC/DEC (1/1)	60 at 50 mA g^−1^	75%@500 cycles (0.1 A g^−1^)	^[^ ^103^ ^]^
	PTCDI‐DAQ	1 m KPF_6_ in DME	209 at 1000 mA g^−1^	73.5%@900 cycles (3 A g^−1^)	^[^ ^14^ ^]^

**TABLE 4:** Summary of the electrolytes on various anodes performance in PIBs

Category	Anode	Electrolyte	Capacity (mAh g^−1^)	Capacity retention	Ref.
Intercalation materials	Graphitic carbon hollow nanocage	1 M KFSI in EC/DEC (1/1)	212 at 0.2 C	92%@100 cycles (0.2 C)	^[9b]^
	Activated carbon	0.8 M KPF_6_ in EC/DEC (1/1)	209 at 100 A g^−1^	62%@100 cycles (0.2 A g^−1^)	^[^ ^104^ ^]^
	F‐doped graphite	0.8 M KPF_6_ in EC/DEC (1/1)	303 at 100 mA g^−1^	74.6%@100 cycles (0.1 A g^−1^)	^[^ ^105^ ^]^
	Graphite fluoride	0.8 M KPF_6_ in EC/DEC (1/1)	281.2 at 100 mA g^−1^	78% @ 50 cycles (0.1 A g^−1^)	^[^ ^106^ ^]^
	Honeycomb‐like N‐doped carbon	0.8 M KPF_6_ in EC/DEC (1/1)	367.1 at 50 mA g^−1^	87.8%@600 cycles (0.3 A g^−1^)	^[^ ^107^ ^]^
Conversion materials	Bismuth‐carbon (Bi@C)	5 M KTFSI in DEGDME	220 at 50 mA g^−1^	85%@600 cycles (0.2 A g^−1^)	^[^ ^108^ ^]^
	Ti_6_O_11_/CNT	0.8 M KPF_6_ in EC/DMC (1/1)	135 at 50 mA g^−1^	76% after 500 cycles (0.2 A g^−1^)	^[^ ^109^ ^]^
	β‐FeOOH	1 M KFSI in EC/DEC (1/1)	218.2 at 100 mA g^−1^	81% after 100 cycles (0.1 A g^−1^)	^[^ ^110^ ^]^
	FeS_2_@NC nanosheets	1 M KFSI in EC/DEC (1/1)	525.5 at 100 A g^−1^	90% after 100 cycles (0.5 A g^−1^)	^[^ ^111^ ^]^
	ZnSe/C	1 M KFSI in EC/DEC (1/1)	318 at 50 mA g^−1^	92% after 1000 cycles (0.5 A g^−1^)	^[^ ^112^ ^]^
Alloying	Sn‐C	0.75 M KPF_6_ in EC/DEC (1/1)	150 at 25 mA g^−1^	81% after 30 cycles (0.025 A g^−1^)	^[^ ^113^ ^]^
	Sb/CNS	1 M KPF_6_ in EC/DMC (1/1)	395.9 at 50 mA g^−1^	90% after 600 cycles (0.2 A g^−1^)	^[^ ^114^ ^]^
	BiSb@C	5 M KFSI in DME	598 at 100 mA g^−1^	97.5% after 600 cycles (0.5 A g^−1^)	^[^ ^115^ ^]^
	BiND/G	1 M KPF_6_ in DME	320 at 100 mA g^−1^	99% after 500 cycles (5 A g^−1^)	^[^ ^116^ ^]^

#### 3.2.1 Development of electrolytes for graphite

According to the electrochemical performance of cathodes and anodes reported, the PIBs still face several challenges. First, the initial columbic efficiency is too low to satisfy the requirement of PIBs full cell. Second, the unstable SEI would accelerate parasitic reactions, then, lead to short low capacity retention and cycle life. Finally, the type of electrolyte limited. Therefore, the development of electrolytes is urgently needed. Graphite is the most used anodes for PIBs, thus, we review recent findings on it to understand the development of electrolytes.

**FIGURE 6:**
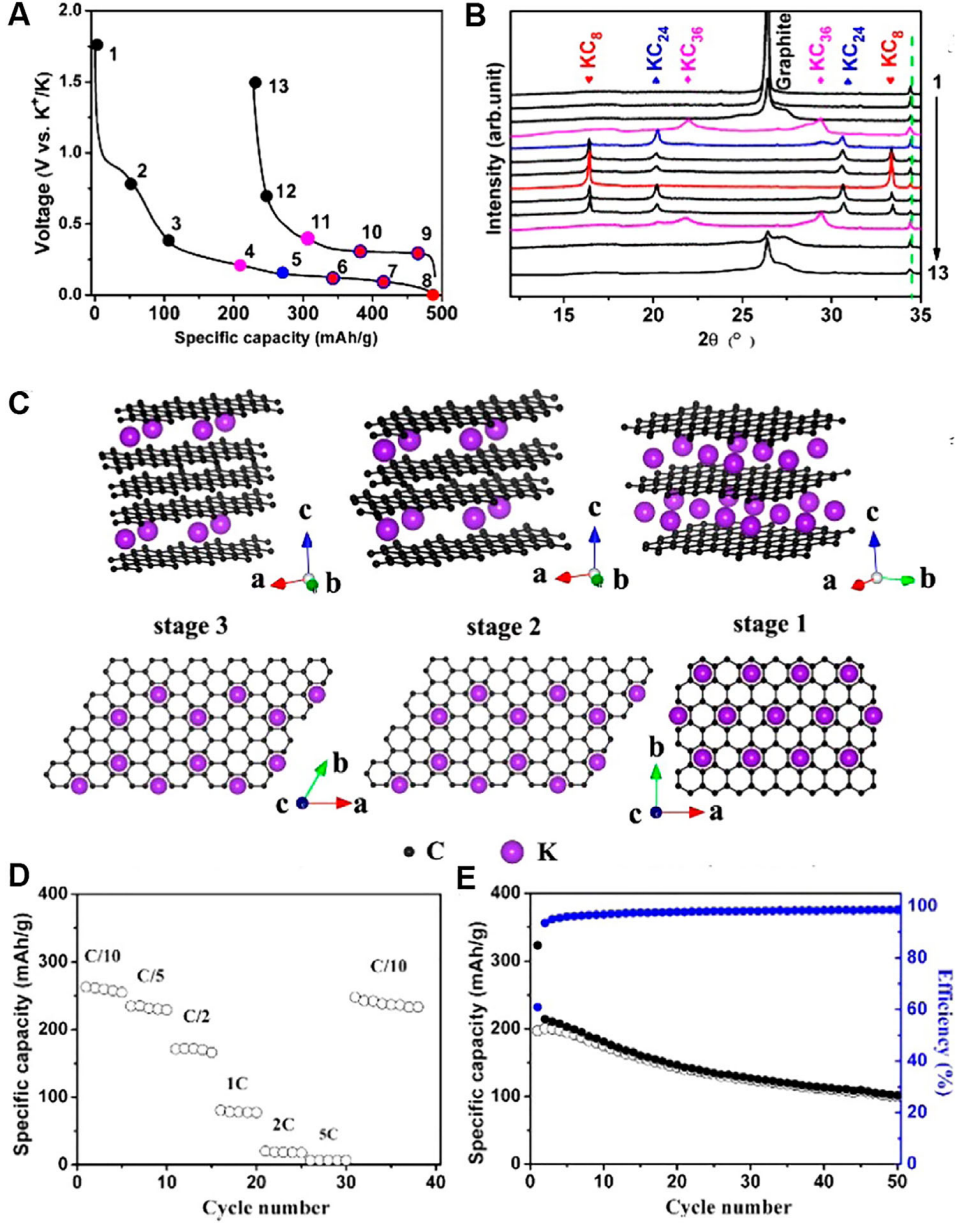
(A) The charge–discharge curve of graphite at C/10. (B) XRD patterns of graphite corresponding to the marked potential in (A). (C) Structure diagrams of different potassium graphite intercalation compounds. (D) Rate performance and (E) cycle performance of graphite at 0.5 C. Reproduced with permission.^[81b]^ Copyright 2015, RSC

Ji et al. first reported the K^+^ electrochemical intercalation behaviors into commercial graphite in 0.8 M KPF_6_ based electrolytes.^[81b]^ They proved potassium graphite intercalation compounds (K‐GIC) have the same structure with that of Li‐GIC (Figure 6A–C). Specially, graphite delivered 273 mAh g^−1^ initial capacity, approaching theoretical value (279 mAh g^−1^) and corresponding the KC_8_ compounds (Figure 6A). Nevertheless, the graphite suffered from limited rate capability and poor cycling stability due to the dramatic volume expansion during charge/discharge process (Figure 6D,E).

**FIGURE 7:**
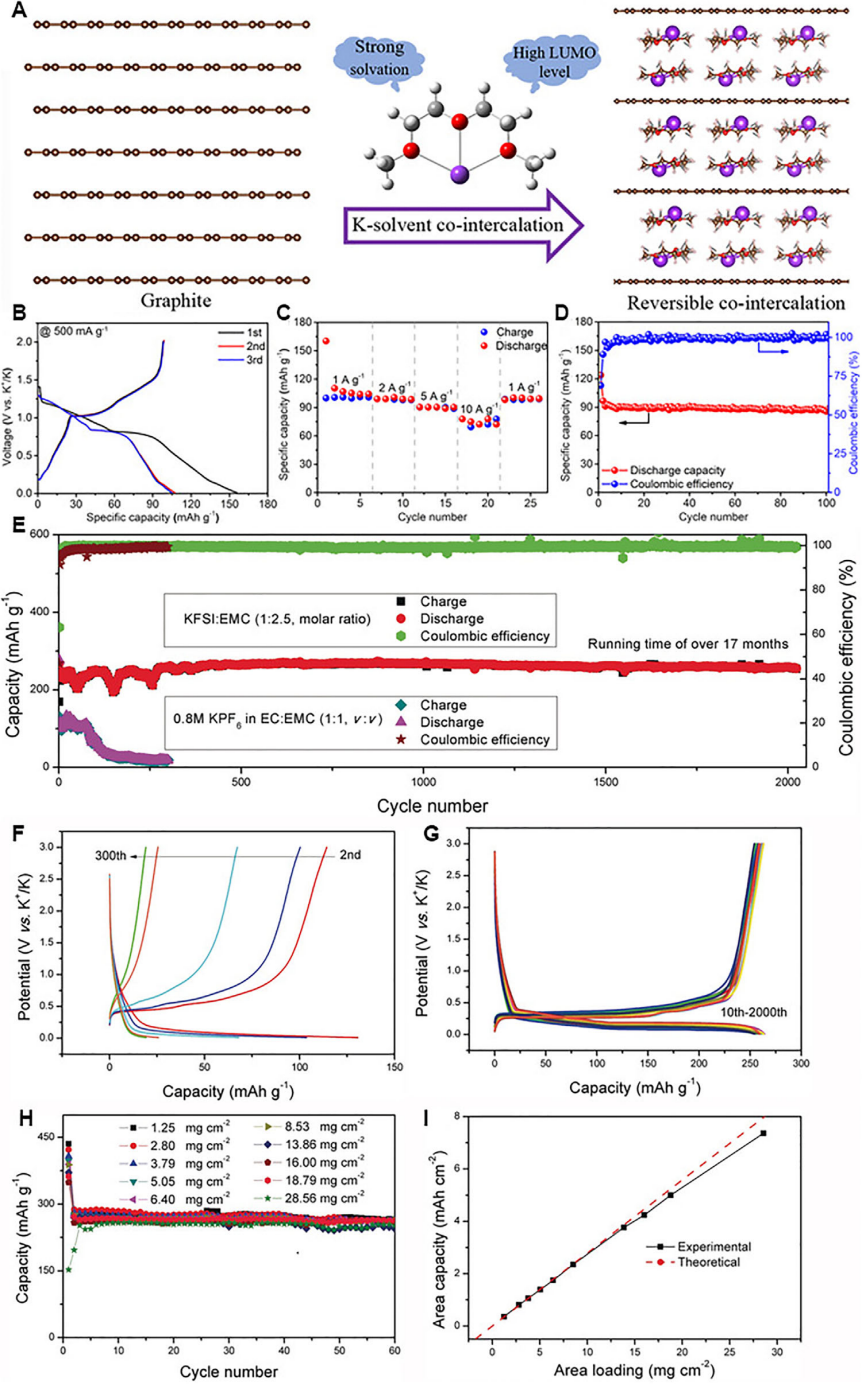
(A) Schematic of reversible K^+^‐solvent co‐intercalation in graphite. (B) Typical charge/discharge curves of graphite electrode based on 1 M KCF_3_SO_3_ DEGDME electrolyte. (C) Rate and (D) cycling performance of the graphite electrode. Reproduced with permission.^[^ ^117^ ^]^ Copyright 2020, Wiley. Electrochemical performance of graphite for potassium storage. (E) Cycling performance of graphite in two different electrolytes. (F,G) Charge–discharge profiles of graphite with 0.8 M KPF_6_ in EC/EMC electrolyte or KFSI/EMC electrolyte. (H) Cycle performance of graphite with different area mass loading in KFSI based electrolyte. (I) Area capacity of graphite calculated from the theoretical and experimental results. Reproduced with permission.^[^ ^118^ ^]^ Copyright 2019, Wiley

To improve the cycle stability of graphite, two type of solvents are adopted. One kind of Solvents is stable enough and could be reversible co‐intercalation into graphite. Or, another class of solvent molecules could decompose to form an effectively stable SEI that restrain the volume change of graphite. Kang et al. systematically explored the intercalation behavior of Li^+^, Na^+^, K^+^ in DEGDME electrolytes and proved ether based electrolytes could maintain great stability and co‐intercalation into graphite.^[^ ^119^ ^]^ Most recently, Chou et al. reported in a unique 1 M KCF_3_SO_3_ based DEGDME electrolyte, graphite exhibited superior K^+^‐solvent co‐intercalation performance (Figure 7A,B).^[^ ^117^ ^]^ Specifically, the graphite showed superior rate performance (77.8 mAh g^−1^ at 10 A g^−1^), maintained 88.5% capacity retention after 100 cycles in PIBs (Figure 7C,D). The co‐intercalation behavior leaves out the desolvation process, thus enabling fast kinetics of K^+^ in SEI. However, the initial coulombic efficiency and reversible capacity of K^+^‐solvent co‐intercalation for graphite are still lower.

Lu et al. explored a high concentrated KFSI/EMC based electrolyte for graphite anode.^[^ ^118^ ^]^ Remarkably, when using KFSI based concentrated electrolyte, the graphite could deliver a stable cycle performance over 2000 cycles with reversible capacity of 255 mAh g^−1^, and the charge–discharge profiles were highly repeatability (Figure 7E–G). The coulombic efficiency of the battery with KFSI based concentrated electrolyte could increase to 99% within 5 cycles. In addition, the graphite electrodes with high mass loadings 28.56 mg cm^−2^ still showed high reversible capacity (Figure 7H,I). Nevertheless, the carbonate or ether‐based electrolytes are highly combustible and volatile, which poses a great potential hazard for explosion or fire accidents. Finding suitable non‐flammable electrolytes represents a tendency for the safe applications of potassium‐based batteries.

**FIGURE 8:**
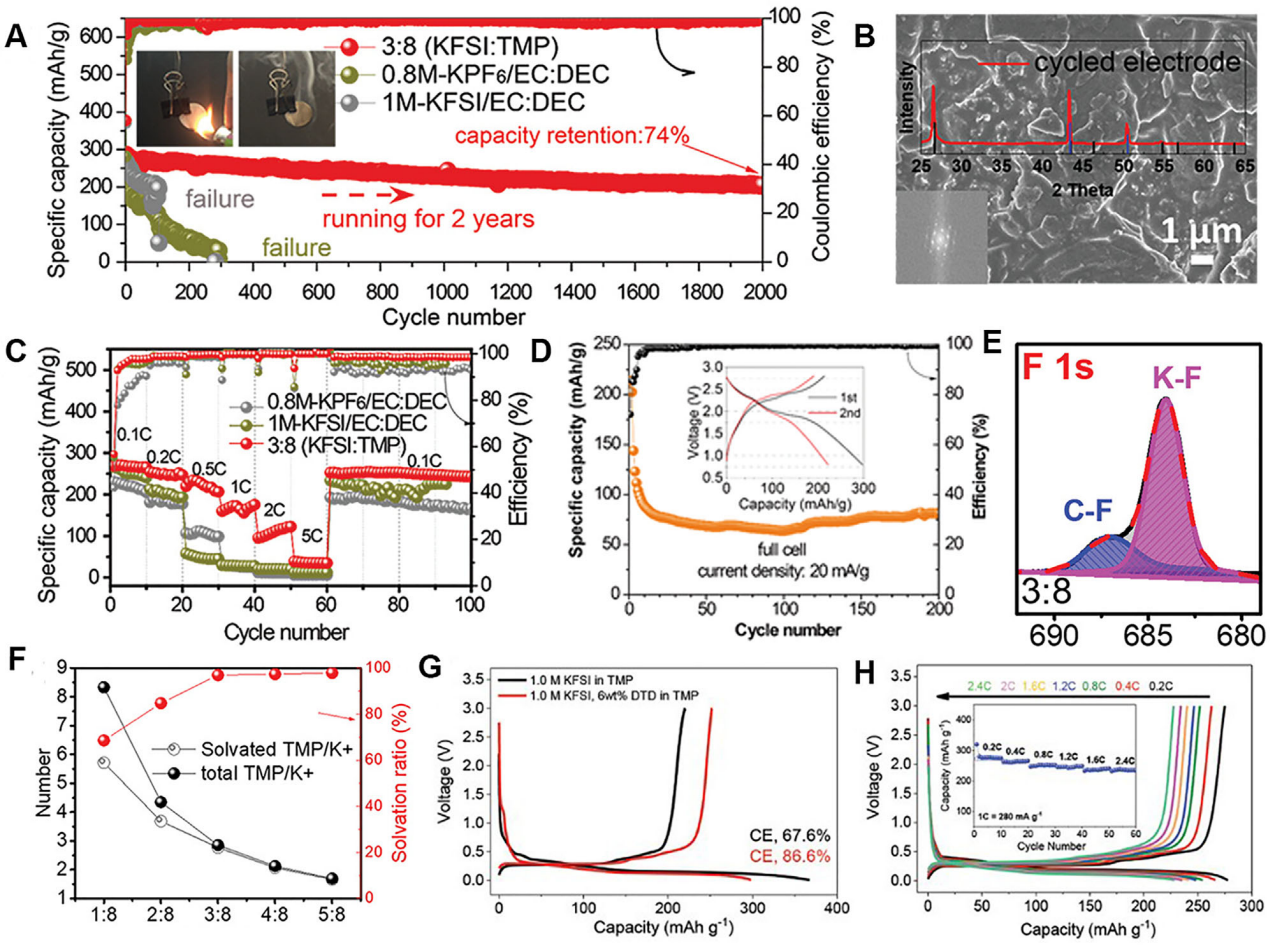
(A) Cycle performance of the graphite electrode based on three different electrolytes, with the inset figures indicating the fire retardance of TMP based electrolyte. (B) SEM image, with the insets showing the SAED and XRD patterns of graphite after 1000 cycles. (C) Rate performance of graphite in three different electrolytes. (D) Cycle performance of graphite||PTCDA full cell TMP based electrolyte at 20 mA g^−1^, with the inset displaying the corresponding first and second cycle charging–discharging profiles. (E) XPS fitting curves of the graphite surfaces after cycling. (F) Solvation ratios, numbers of TMP with K^+^, and total numbers of TMP molecules per K^+^ ion at different molar ratios. Reproduced with permission.^[^ ^57^ ^]^ Copyright 2021, Wiley. (G) Comparative first (dis‐)charge curve of graphite electrode in the electrolyte of 1.0 M KFSI in TMP with and without DTD. (H) Rate capability of graphite electrode in the electrolyte with 6 wt% DTD. Reproduced with permission.^[^ ^77^ ^]^ Copyright 2020, Wiley

Phosphorus‐based organic solvents are often used as nonflammable additives to retard possible fire hazard in LIBs. Guo et al. made attempts to use TEP as single solvent in graphite/K_0.5_MnO_2_ K ion cells, and found the cell showed high electrochemical performance.^[^ ^120^ ^]^ Subsequently, Guo and Ming et al. improved the compatibility of phosphorus‐based solvents with graphite anodes through adjusting solvent, concentration, and additives.^[^ ^57^, ^77^ ^]^ The commercial graphite in moderate‐concentration KFSI‐TMP based electrolyte maintained unprecedented cycle stability with 74% capacity retention over 2000 cycles (Figure 8A). This performance was significantly superior to the conventional EC/DEC electrolytes with either KPF_6_ or KFSI salt. In addition, the graphite electrode still maintained its integrity after cycles with its SEI film coverage intact, as shown in Figure 8B. The excellent rate and full battery (graphite||perylenetetracarboxylic dianhydride) performance was also demonstrated (Figure 8C,D). Electrolyte structure and surface analyses (Figure 8E,F) showed that the nearly 100% solvation of TMP with K^+^ and FSI^−^‐derived F‐rich SEI could effectively suppress the reduction of the solvents. The ethylene sulfate (DTD) additive further made TMP based electrolyte more compatible with graphite anode. The graphite showed a high initial CE and rate capability in such a newly designed electrolyte with 6 wt% DTD (Figure 8G,H).

Currently, novel fire‐retardant ionic liquids (ILs) have been developed for high performance PIBs. They exhibit excellent physical properties, such as K[FSI]‐[C3C1pyrr][FSI] (C3C1pyrr = *N*‐methyl‐*N*‐propylpyrrolidinium) with high ionic conductivity and wide electrochemical window (4.8 mS cm^−1^ and 5.72 V, respectively),^[^ ^121^ ^]^ K single cation IL (K‐ SCIL, K[FSI]_0.55_[FTA]_0.45_) with high K^+^ transport number^[^ ^122^ ^]^ and good compatibility with graphite anode. Nevertheless, the understanding of interface between graphite and ILs is not as mature as the LIBs/SIBs, and the application of ILs in PIBs is in the initial stage. Despite some breakthroughs in electrolytes for graphite have been made, the electrolyte designing for various cathode materials and full batteries are scarce.

#### 3.2.2 Development of electrolytes for Prussian blue analogues

So far, PBAs have been widely used as cathodes for PIBs. They belong to metal hexacyanoferrates family with composition of A*_x_* M_A_[M_B_ (CN)_6_]*_y_* ·*z*H_2_O, where M_A_ and M_B_ are transition metals (e.g., Co, Fe, Mn, Ni), A is alkali metal (e.g., Li, Na, K), and x ranges from 0 to 2 depending on the valance of M_A_ and M_B_.^[^ ^123^ ^]^ The first research on the activity of KFe[Fe(CN)_6_] for K^+^ storage capacity was reported in 2004,^[^ ^124^ ^]^ and the reaction mechanism was proposed in 1 M KBF_4_ based EC/EMC electrolytes. However, the low specific capacity is difficult to meet demand. Therefore, K_1.89_Mn[Fe(CN)_6_]_0.92_·0.75H_2_O as a cathode for KIBs was proposed.^[^ ^125^ ^]^ This material showed experimental capacity of 142 mAh g^−1^ in KClO_4_ salt based electrolyte, close to the theoretical discharge capacity of 156 mAh g^−1^. However, it experienced a large voltage polarization (>0.5 V) due to low the ionic conductivity induced by low concentration of KClO_4_ salt. However, it showed high polarization during charging and discharging process. Later, Komaba et al. demonstrated higher discharge plateaus for the same compound in KPF_6_ based EC/DEC electrolytes.^[^ ^73^ ^]^ However, the higher voltage could reduce the stability of electrolyte and accelerate the solubility of transition metals. Therefore, the high voltage resistance and interface stability electrolyte is the future development direction, such as, local high concentration electrolytes (LHCEs), ionic liquid, molten salt electrolytes, and solid electrolytes.

### 3.3 Liquid electrolytes for K metal batteries

#### 3.3.1 Potassium metal batteries

**FIGURE 9:**
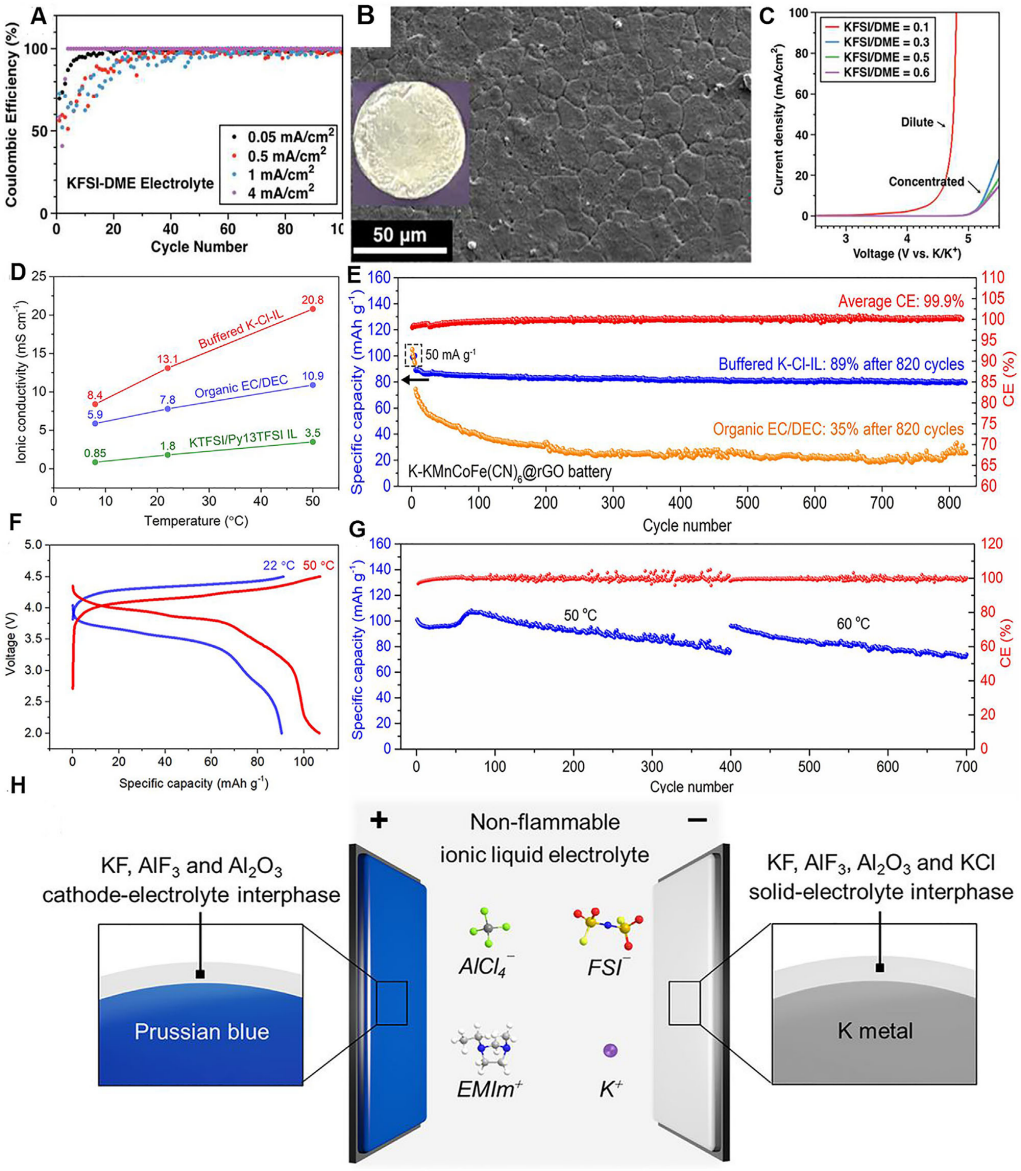
(A) cycling performance of K metal in KFSI/DME electrolytes. (B) SEM imaging of the plated K in KFSI/DME electrolyte. (C) Electrochemical stability of KFSI/DME electrolytes. Reproduced with permission.^[^ ^126^ ^]^ Copyright 2017, RSC. (D) Ionic conductivities of the buffered K‐Cl‐IL, 0.9 M KPF_6_ in EC/DEC (1/1), and 0.5 M KTFSI in Py_13_TFSI IL at 8, 22, and 50°C. (E) Cycling performances of K metal‐KMCFC@rGO batteries using in K‐Cl‐IL and organic electrolytes at 100 mA g^−1^ with cycling at 50 mA g^−1^ for five cycles at first marked by the dashed rectangle. (F) Charge/discharge curves of K metal‐KMCFC@rGO battery running at 22 and 50°C. Current density, 100 mA g^−1^. (G) Cycle performance of K metal‐KMCFC@rGO battery at 50 and 60°C. Current density, 100 mA g^−1^. (H) Schematic diagram of the battery configuration, electrolyte composition, and SEI component. Reproduced with permission.^[^ ^127^ ^]^ Copyright 2020, NAS

**FIGURE 10:**
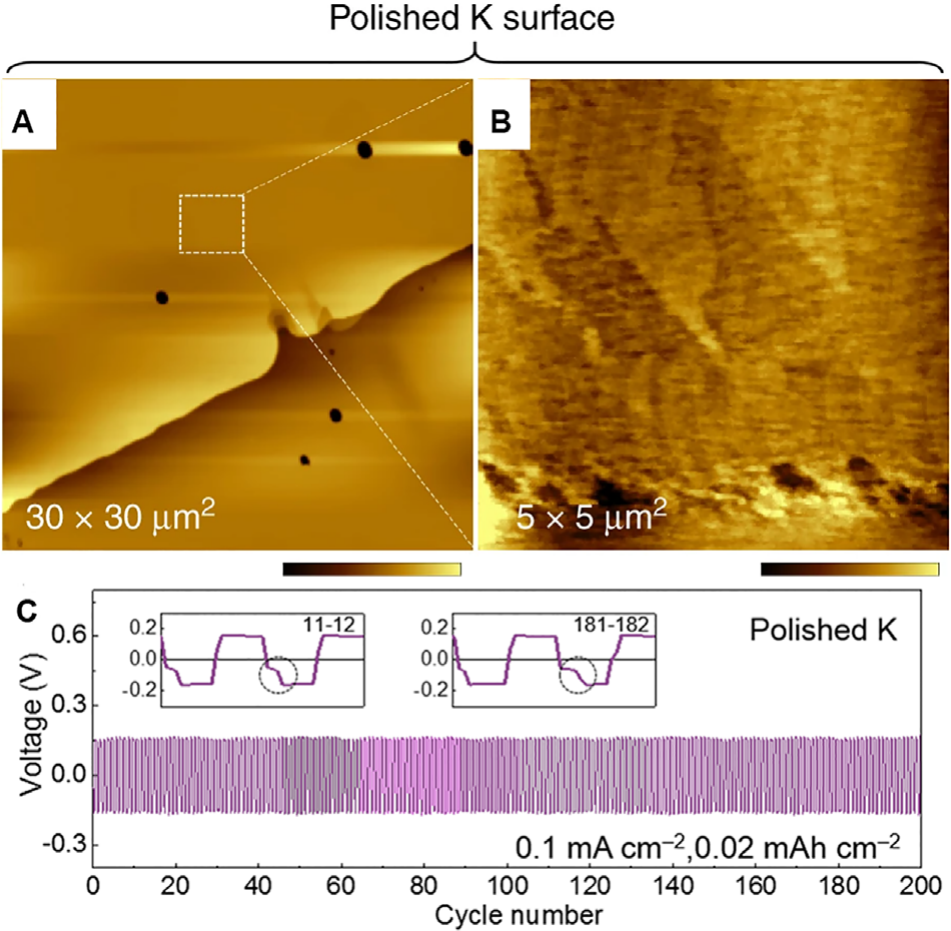
(A,B) AFM images of polished K surface. (C) Voltage profiles of symmetric polished K cells. Reproduced with permission.^[^ ^130^ ^]^ Copyright 2018, Springer Nature

K metal anode received extensive attention as anode for high‐capacity KMBs. However, the ultrahigh reactivity resulting unstable SEI film, dendrites growth, and huge volume change severely constrain its application.^[^ ^128^ ^]^ Considerable efforts have been made to solve these problems, such as, constructing various conductive hosts and artificial SEI, developing current‐controlled and modifying electrolytes.^[^ ^129^ ^]^ The morphological characteristics and structural stability of SEI film play an important role in the capacity, power, cycle life, and high temperature stability of battery. Wu et al. first reported high concentrated KFSI/DME electrolyte could enable reversible potassium plating/stripping (Figure 9A).^[^ ^126^ ^]^ The electrochemically deposited K metal presented a flat block morphology with an average size of 15 μm (Figure 9B), which attributed to the compactness of SEI. Furthermore, the KFSI/DME electrolyte showed wide voltage stability window over 5 V (Figure 9C). Recently, Dai et al. also reported a nonflammable ionic liquid electrolyte consisting of 1‐ethyl‐3‐methylimidazoliumchloride/AlCl_3_/KCl/KFSI (K‐Cl‐IL) for safe and high‐capacity KMBs.^[^ ^127^ ^]^ It showed high ionic conductivity of 13.1 mS cm^−1^ at 22 (Figure 9D). A battery with Prussian blue/reduced graphene oxide (KMCFC@rGO) as cathode in K‐Cl‐IL electrolyte showed a long cycling stability over 820 cycles, with high retaining rate (≈89%) and CE (≈99.9%), as show in Figure 9E. Furthermore, the K‐Cl‐IL electrolyte enabled K battery work well above room temperature (Figure 9F,G). The author attributed high cycling stability of K metal batteries to the robust passivation layers rich in K, Al, F, and Cl‐based species (Figure 9H). Mao et al. also verified the electrochemical polishing method could design high‐performance K metal batteries in 1 M KTFSI/DME electrolyte.^[^ ^130^ ^]^ Remarkably, the SEI layers on K metals were ultra‐flat with molecular‐scale roughness (Figure 10A). The symmetric cells with two polished K metal electrodes showed enhanced stability of at least 200 cycles (Figure 10B).

**FIGURE 11:**
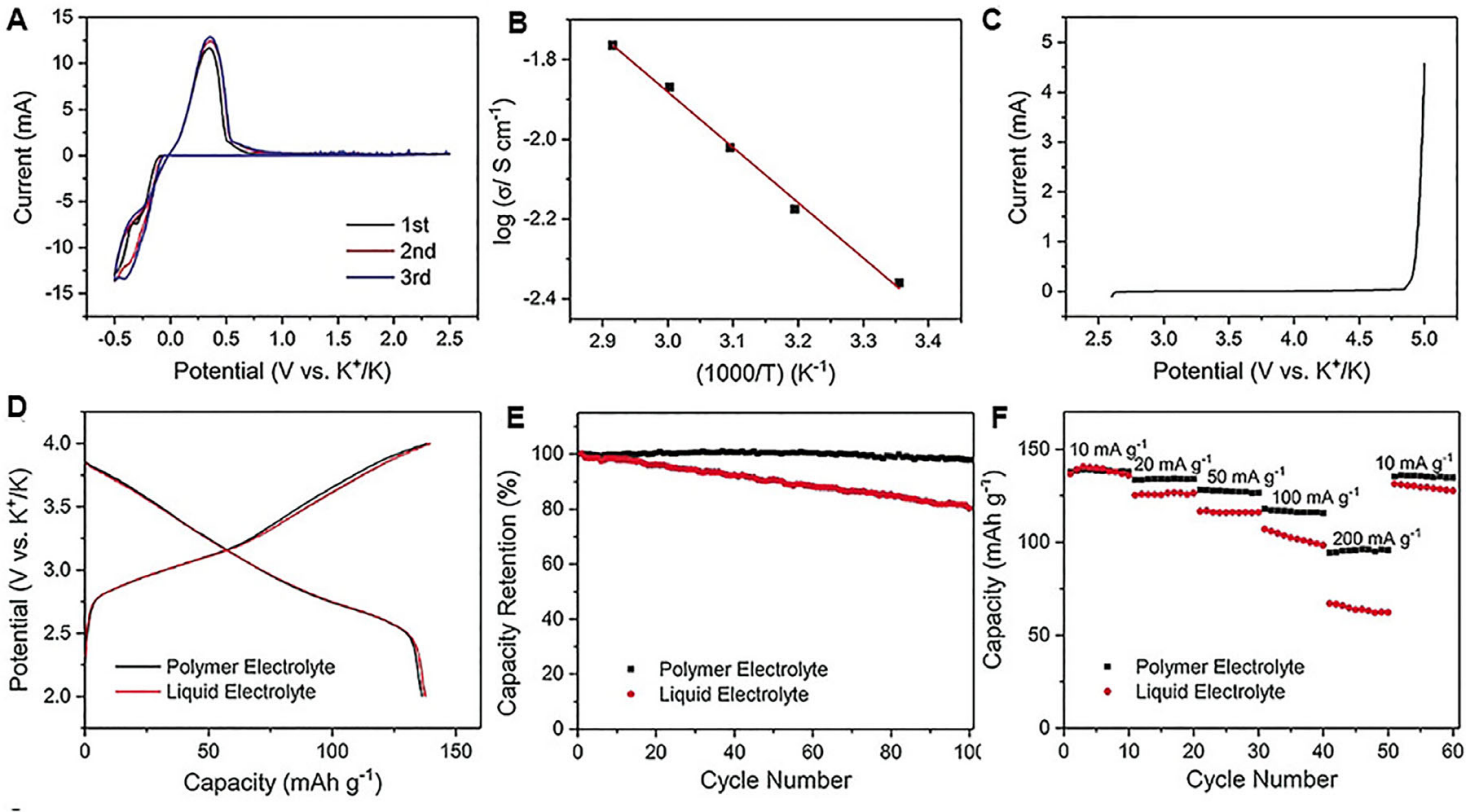
(A) The plating/striping behavior of potassium in a coin cell with the polymer‐gel electrolyte at 1.0 mV s^−1^. (B) Ionic conductivity of the PMMA polymer‐gel electrolyte with temperature. (C) LSV plot of PMMA at 1.0 mV s^−1^. (D) Charge/discharge curves of the polyaniline cathode at 10 mA g^−1^. (E) Cycle performance of the polyaniline cathode at 50 mA g^−1^. (F) Rate performance of the polyaniline cathode ranging from 10 to 200 mA g^−1^. Reproduced with permission.^[^ ^132^ ^]^ Copyright 2018, Wiley

GPE has also been investigated with growing interest because of combinative of liquid and solid‐state electrolytes. Jyothi et al. demonstrated polyacrylonitrile (PAN)/potassium iodide (KI) GPE with conductivity of 2.089 × 10^−5^ S cm^−1^ at 30°C and activation energy of 0.358 eV.^[^ ^131^ ^]^ Gao et al. first reported the application of poly(methyl methacrylate) (PMMA) GPE in K||polyaniline cell.^[^ ^132^ ^]^ The GPE with high conductivity of 4.3 × 10^−3^ S cm^−1^ realized reversible plating/stripping of K at room temperature (Figure 11A,B). Moreover, the strong oxidation resistance over 4.9 V is conducive to match with high‐voltage cathodes (Figure 11C). The K||polyaniline cell with PMMA exhibited nearly the same discharge capacity (138 mAh g^−1^) as the cell with the organic liquid electrolyte at 10 mA g^−1^ (Figure 11D), and even a better cycle and rate performance (Figure 11E,F). Recently, Tang et al. prepared a flexible Sn||AC potassium ion hybrid capacitor based on 3D porous poly(vinylidene fluoridehexafluoropropylene) (PVDF‐HFP)/KPF_6_ GPE.^[^ ^133^ ^]^ The GPEs not only improved the structural stability of the Sn anode, achieved long cycling stability, but also realized good flexibility.

In addition, several notable successes have achieved for stable K plating/stripping based on regulating the nucleation and deposition of K ions. For example, infiltrated carbon nanotube membranes, functionalized 3D copper, and puffed millet/NiO current collectors, Na‐K alloy and Sn‐based solid‐electrolyte interphase were developed to stabilize the metal front.^[^ ^134^ ^]^ However, the customized electrolytes for K‐based batteries are far behind lithium and sodium metal. Thus, in this context, the research of electrolyte materials and science, especially the fundamental understanding of interfacial processes, constitutes the key areas for future K ions storage.

### 3.4 Liquid electrolytes for K‐S/Se/O_2_ batteries

#### 3.4.1 K‐S batteries

Like (Li, Na)‐sulfur batteries, potassium‐sulfur (K‐S) batteries has a theoretical energy density of 914 Wh kg^−1^, much higher than the current commercial lithium‐ion battery, which is a key motivation for further study.^[^ ^135^ ^]^ The high abundance and low cost of sulfur further make it a great potential for the next generation of rechargeable batteries.^[^ ^136^ ^]^ Alternatively, Se cathode with remarkable electrical conductivity of 1.0 × 10^−3^ S cm^−1^ and comparable theoretical volumetric capacity, is also a promising cathode material.^[^ ^137^ ^]^ Therefore, K‐S/Se batteries are considered as ideal choices for the next generation of high energy density battery system. However, the research of K‐S batteries system is still in the preliminary stage and faces a series of challenges including the electrochemistry of sulfur and sulfide/polysulfide, K metal anode, compatible electrolyte, as well as, the overall aspects of full‐cell.

**FIGURE 12:**
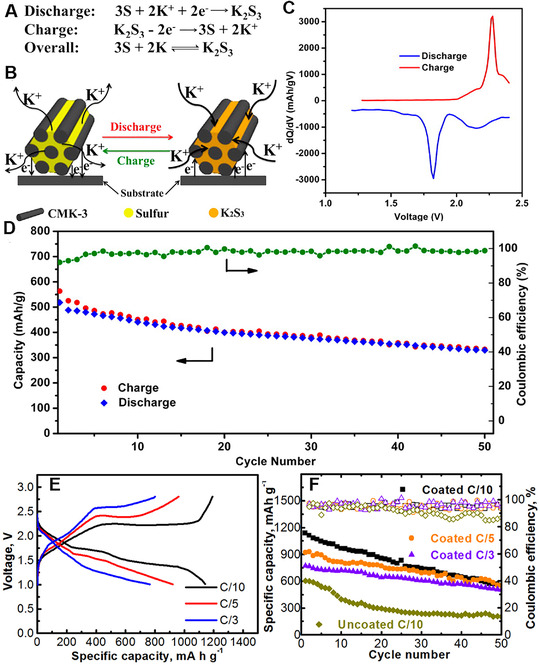
(A) Discharge, charge, and overall reaction. (B) Schematic diagram of K‐S batteries with CMK‐3/sulfur electrode. (C) Corresponding differential capacity plots of S cathode. (D) Cycling performance and coulombic efficiency at 50 mA g^–1^. Reproduced with permission.^[135a]^ Copyright 2014, RSC. (E) Discharge/charge profiles of the KǁCelgard/SWCNTǁS/CNF cells at various rates. (F) Cycling performance and CE with cycle number for the KǁCelgard/SWCNTǁS/CNF cells and the KǁCelgardǁS/CNF cell. Reproduced with permission.^[^ ^138^ ^]^ Copyright 2018, Elsevier

Chen et al. first designed rechargeable K‐S batteries using ordered mesoporous carbon (CMK‐3)/sulfur composites as the cathode in TEGDME based electrolyte (Figure 12A,B).^[135a]^ The S cathode showed a sloping region and a platform at about 1.8 V in the ether based electrolyte (Figure 12C). However, the initial discharge capacity was as low as 523.5 mAh g^−1^, and reduced to 329.3 mAh g^–1^ over 50 cycles (Figure 12D). To enable deep discharge–charge of sulfur cathode, Manthiram et al. rationally designed a cathode separator in 1 M KCF_3_SO_3_/TEGDME electrolyte, which greatly improved the discharge capacities of room‐temperature K‐S cells (Figure 12E).^[^ ^138^ ^]^ After 50 continuous cycles, the discharge capacity remained at ≈600 mA h g^−1^ (Figure 12F).

**FIGURE 13:**
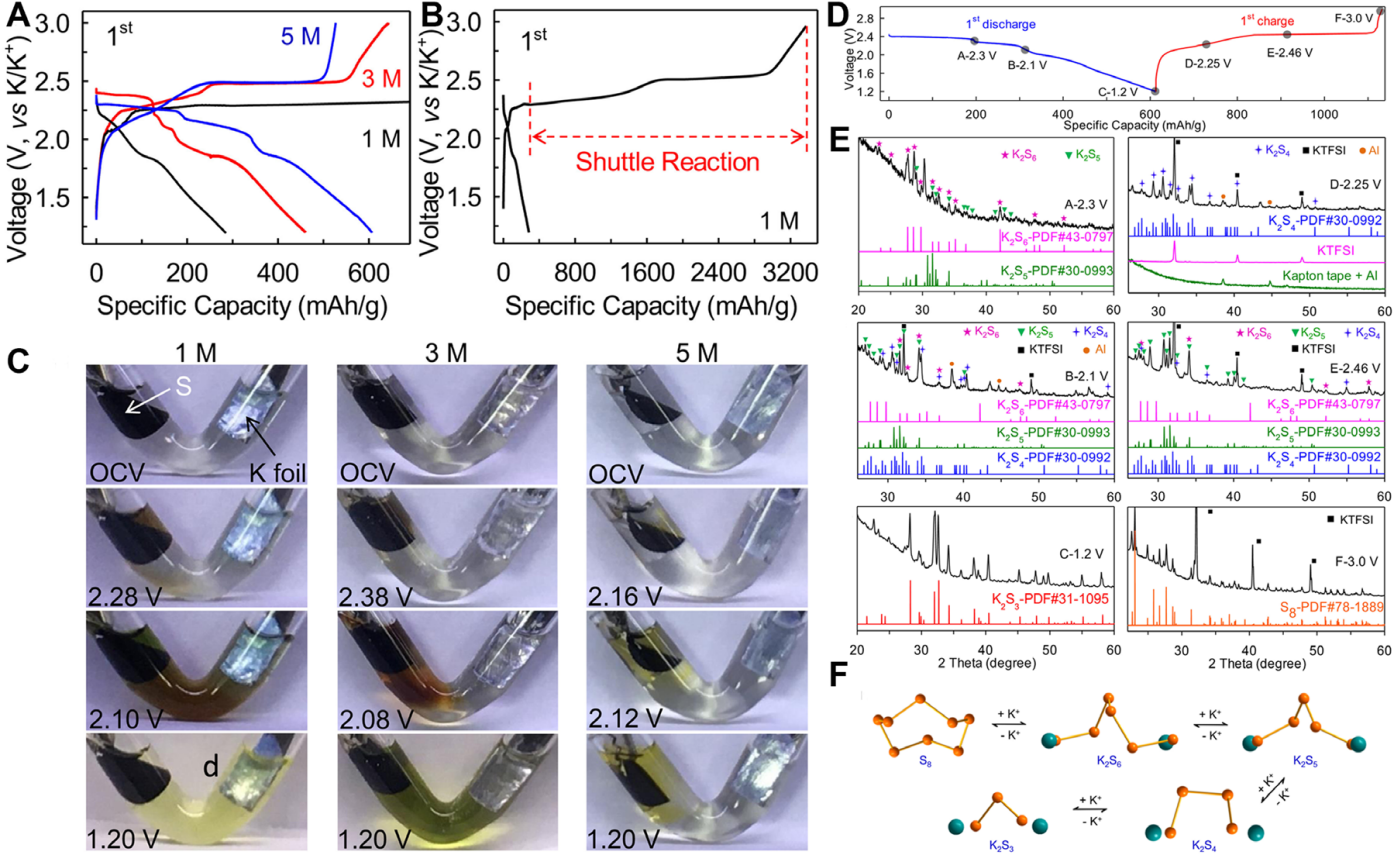
(A,B) First‐cycle discharge/charge profiles of K‐S batteries at 10 mA g^−1^. (C) “Transparent” batteries capture the polysulfide shuttle behaviors. (D)Voltage profile at 5 mA g^−1^. (E) Ex‐situ XRD patterns. (F) Schematic showing the reaction mechanism interpreted by XRD analysis. Elsevier. Reproduced with permission.^[^ ^139^ ^]^ Copyright 2019, Elsevier

The continuous dissolution, relocation, and shuttle of polysulfides lead to the low utilization rate of active materials and passivation of positive/negative electrodes, which further result in rapid capacity decay and low CE. To counter the shuttle effect, Sun et al. first demonstrated that high concentrated electrolyte could effectively mitigate this parasitic effect and enabled a full energy utilization.^[^ ^139^ ^]^ In the 5 M KTFSI/TEGDME electrolyte, the K‐S battery delivered an initial specific capacity of 606 mAh g^−1^ and a reversible specific capacity (527 mAh g^−1^) with an initial CE of 86.96% (Figure 13A). While, a ≈11 times higher initial charge capacity (3375 mAh g^−1^_S_) than discharge capacity of 285 mAh g^−1^_S_ was observed in 1 M electrolyte, which attributed to severe dissolution of polysulfide intermediates (Figure 13B). To visualize the concentration effect of electrolyte, “transparent” batteries were fabricated (Figure 13C). It showed the colorless 1 M electrolyte turned dark brown on the cathode side upon discharged to 2.28 V, and this brown color spread all over the electrolyte upon further discharged to 2.10 V. These observations evidently confirmed that the potassium polysulfide intermediates dissolved in the electrolyte and shuttled to the anode side. As the reaction proceeded, the clear electrolyte changed to a light yellow turbid liquid at 1.20 V, demonstrating that the soluble polysulfide intermediates were chemically reduced by K metal into insoluble species. This shuttle phenomenon remained severe in the 3 M electrolyte, not as serious as in the 1 M one. Surprisingly, it was observed that only the electrolyte surrounding the S cathode displayed light yellow in 5 M electrolyte, whereas the solution on the anode side remained colorless even at 1.20 V. Furthermore, they provided the first unambiguous evidence about reversible stepwise phase transformations of S_8_ ⇔K_2_S_6_ ⇔K_2_S_5_ ⇔K_2_S_4_ ⇔K_2_S_3_ by X‐ray powder diffraction analysis (Figure 13D–F). In order to solve address the problem of fast active‐material loss, more recent researches focus on confining sulfur within the porous carbon host, chemical binding sulfur, and combining homogeneous catalyst.^[^ ^82^, ^140^ ^]^

#### 3.4.2 K‐Se batteries

**FIGURE 14:**
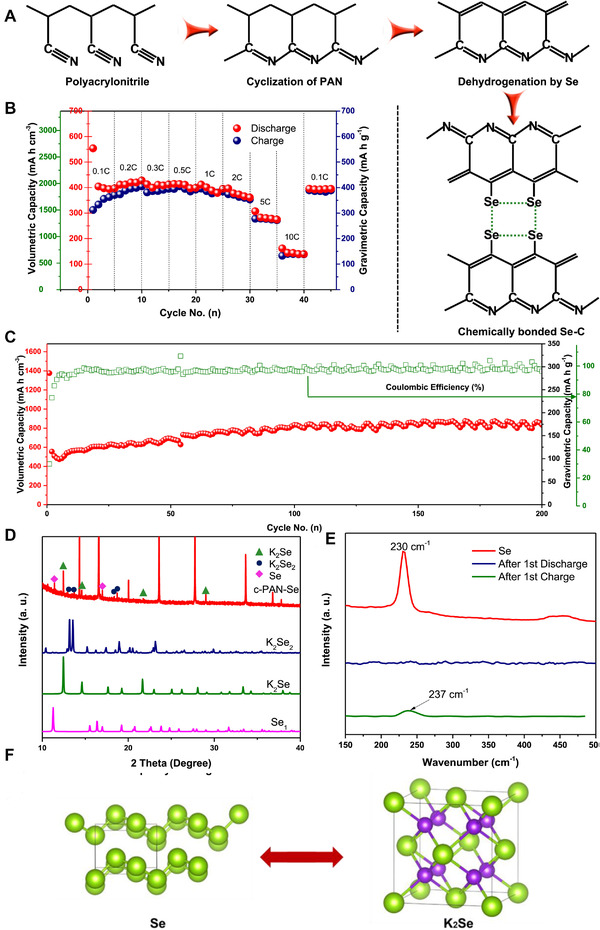
(A) Reaction mechanism of c‐PAN‐Se. (B) Rate capabilities of c‐PAN‐Se. (C) Cycle performance of c‐PAN‐Se at 5 C. (D) HEXRD pattern of c‐PAN‐Se. (E) Raman spectroscopic analysis of c‐PAN‐Se. (F) Structural diagram of the Se and K_2_Se structures. Reproduced with permission.^[^ ^141^ ^]^ Copyright 2017, Elsevier

Guo et al. first reported a reversible K‐Se battery with selenium/carbonized‐PAN composite (c‐PAN‐Se) as cathode in 1 M KFP_6_ (EC/PC = 1:1) electrolytes (Figure 14A).^[^ ^141^ ^]^ The as‐prepared K‐Se battery showed high cycling and rate performance (Figure 14B,C). The c‐PAN‐Se electrode maintained capacity of 89% at 0.2 C compared to the second cycle capacity at 0.1 C. Even at 10 C, it still could deliver a capacity of 673 mAh cm^−3^. The capacity of 835 mAh cm^−3^ could be retained after 200 cycles with CEs up to 99%. Moreover, the reversible Se + 2K⇔ K_2_Se one‐step conversion reaction in K‐Se cells was proposed with the aid of high energy XRD (HEXRD) and Raman characterization (Figure 14D–F).

**FIGURE 15:**
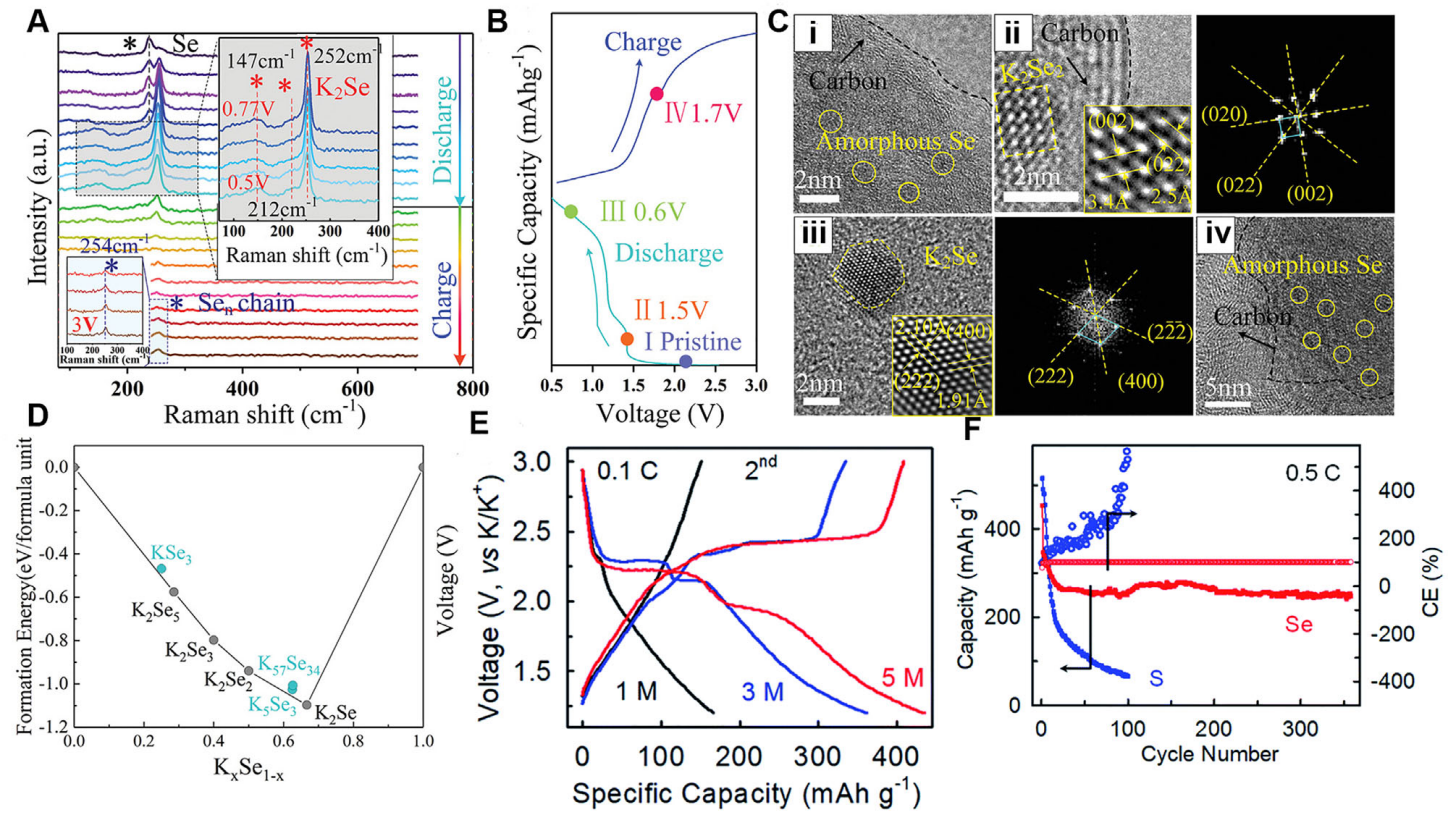
(A) In situ Raman analysis of the Se@NPCFs in the K‐Se batteries at different voltages. (B,C) Ex situ HRTEM images of the Se@NPCFs electrode from the left discharge/charge curve at different states. (D) Formation energies of existing potassiation selenides by theoretical calculations. Reproduced with permission.^[^ ^142^ ^]^ Copyright 2020, Wiley. (E) Voltage curves of the K‐Se batteries in 1, 3, and 5 M electrolytes. (F) Cycle performance of the K‐Se batteries in 5 M electrolytes. Reproduced with permission.^[^ ^143^ ^]^ Copyright 2020, ACS

Subsequently, it was proved that the Se cathode experienced a two‐step reduction mechanism by Yu and coworkers in 0.7 M KPF_6_ based ester electrolyte.^[^ ^142^ ^]^ As shown in Figure 15A, in situ Raman spectroscopy were conducted to characterize the changes of Se during the initial CV measurement. A trigonal Se peak was in gradual transition to 252 cm^−1^ representing K_2_Se during discharge, and the produced K_2_Se was fully oxidized to chain‐like Se molecules again in the charge processing. The obtained HRTEM images, intercepted at different states during discharge–charge, showed that Se_2_ molecules were initially converted into K_2_Se_2_ and then reduced to the final product K_2_Se (Figure 15B,C). The DFT calculation (Figure 15D) further indicated that multi stable structures exhibited on the convex process. The stepwise phase transformation made K‐Se batteries suffer from the detrimental polyselenides dissolution, shuttle reactions, and slow dynamic conversion process. To solve the problem, Sun et al. developed a K‐Se battery in concentrated ether‐based electrolytes, which displayed a capacity of 252 mAh g^−1^_Se_ after 350 cycles at 0.5 C with average discharge plateau voltage of 1.85 V (Figure 15E,F).^[^ ^143^ ^]^ However, only a few electrolytes exhibit impressive gravimetric and volumetric capacities.^[^ ^144^ ^]^ Thus, exploring novel strategies, involving organic solvent, appropriate concentration, all‐solid‐state electrolytes, modified cathode, and deeply understanding the redox mechanisms, are deeply needed for K‐Se batteries.

#### 3.4.3 K‐O_2_ batteries

**FIGURE 16:**
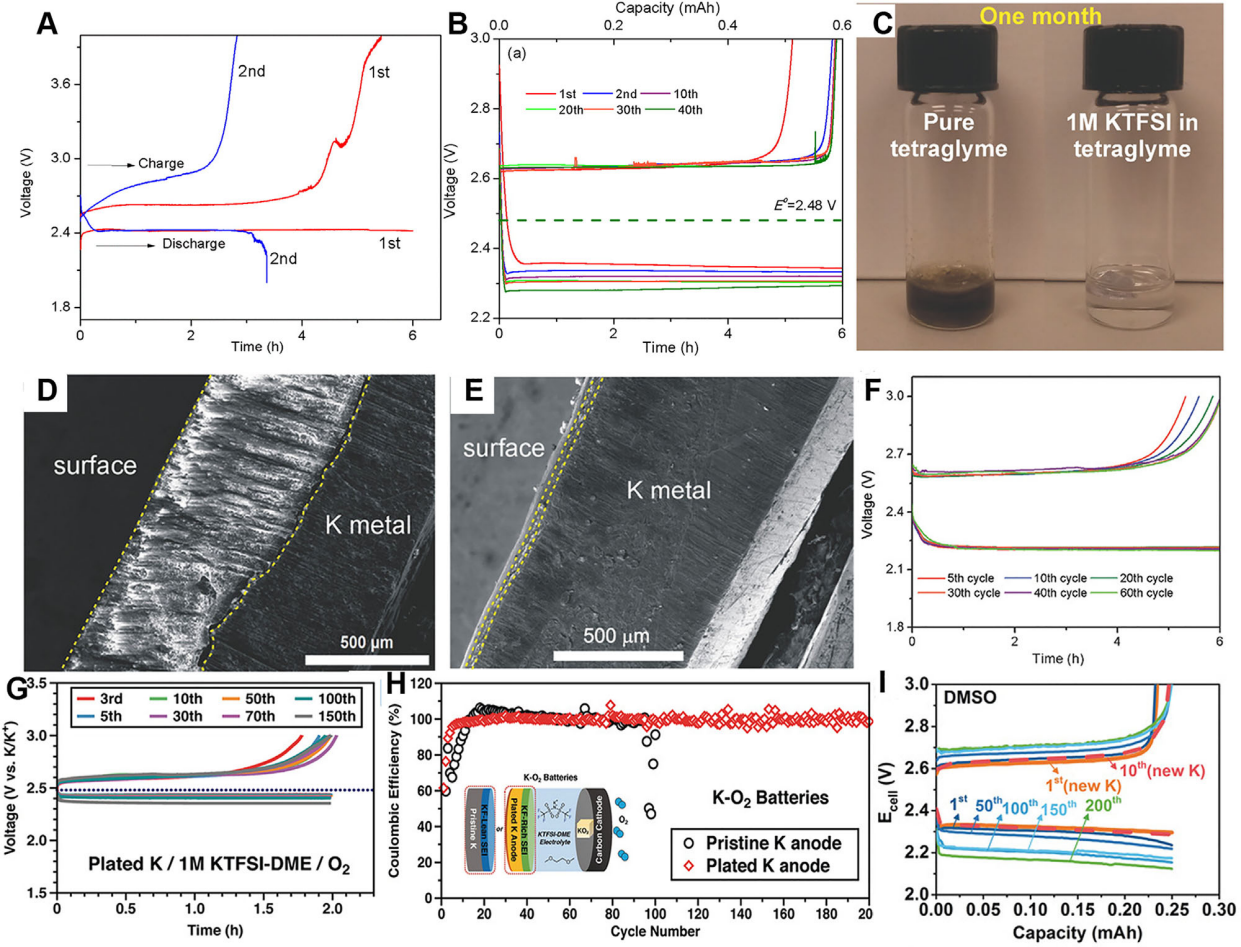
(A) The first two continuous battery discharge–charge cycles at 0.16 mA cm^−2^ current density. Reproduced with permission.^[^ ^146^ ^]^ Copyright 2013, RSC. (B) Discharge/charge profiles of K‐O_2_ batteries with a Nafion‐K^+^ membrane. Reproduced with permission.^[^ ^147^ ^]^ Copyright 2014, ACS. (C) The stability test of K metal with pure tetraglyme and 1 m KTFSI in tetraglyme under Ar after one month. (D,E) Cross‐section SEM of the K anode in K‐O_2_ battery with 1 M KPF_6_, and 1 M KTFSI based DME electrolyte, respectively. (F) Voltage curves of K‐O_2_ battery with 1 M KTFSI based DME electrolyte. Reproduced with permission.^[^ ^148^ ^]^ Copyright 2017, Wiley. (G) Voltage curves of K‐O_2_ battery with the modified K anode. (H) Cycle performance of K‐O_2_ cells with pristine and modified K metal anodes (cycling capacity: 0.056 mAh cm^−2^). Reproduced with permission.^[^ ^149^ ^]^ Copyright 2018, Wiley. (I) Voltage curves of DMSO‐based K‐O_2_ batteries. Reproduced with permission.^[^ ^150^ ^]^ Copyright 2018, Wiley

In addition, the facile one‐electron redox process of K + O_2_ + e‐↔ KO_2_ solves the major challenges in Li‐O_2_ batteries. The thermodynamically and kinetically stable KO_2_ redoubles the advantage of K‐O_2_ batteries.^[^ ^145^ ^]^ Wu et al. first demonstrated the concept of K‐O_2_ battery with low overpotential by taking advantage of the reversibility of the O_2_/O^2−^ redox couple in 0.5 M KPF_6_ ether electrolyte (Figure 16A).^[^ ^146^ ^]^ The highly reductive potassium metal, and the crossover of O_2_ molecules to the anode, along with severe side reaction, also had a substantial impact on cycling performance of K‐O_2_ batteries. To alleviate parasitic reactions, the polymeric K^+^ membrane (Nafion‐K^+^) was applied as K‐O_2_ battery separator, which improved the cycle life over 40 cycles (Figure 16B).^[^ ^147^ ^]^ Ren et al. developed a solvent‐ and O_2_‐impermeable layer by applying KTFSI based ether electrolyte.^[^ ^148^ ^]^ First, K metal undissolved in 1 M KTFSI tetraglyme electrolyte, and no blue color was observed and the electrolyte was still clear after one month (Figure 16C). Second, the surface layer formed in KTFSI‐based electrolyte (2–3 μm) was much thinner than that of in KPF_6_ electrolyte (500 μm) (Figure 16D,E), and also an effective O_2_ barrier. The layer was mainly composed of the decomposition products derived from TFSI^−^ anions, accompanied by a small quantity of DME reduction products. As a result, K‐O_2_ batteries showed significantly improved cycling stability over 60 cycles (Figure 16F).

However, the three‐electrode measurement showed the SEI in KTFSI base electrolyte had low K^+^ conductivity, increasing K plating/stripping overpotential. Wu et al. developed a modified SEI in KFSI‐DME electrolyte for K metal anode.^[^ ^149^ ^]^ The modified K anode showed significantly reduced the overpotential and doubled the cycle life of K‐O_2_ batteries in KTFSI based electrolyte (Figure 16G,H). Apart from achieving stable K anode, improving the reversibility of KO_2_ is vital for the application of K‐O_2_ batteries. Lu et al. exploited a strong electron‐donating DMSO solvent to stabilize superoxide, resulting in significant improvement in electrode kinetics and cycle life (Figure 16I).^[^ ^150^ ^]^ Therefore, the studies on the stabilization of K‐metal anodes and/or the functionalization of electrolyte solutions for K‐O_2_ batteries are urgent needed.

## 4 SOLID‐STATE ELECTROLYTES

In the previous chapters, the recent progress of widely applied organic electrolytes has been reviewed. However, the high flammability and volatility of organic electrolytes raises the safety concern of KIBs. Therefore, the development of solid electrolytes (SSEs) has become a hot issue with great potential for both academia and industry. Compared to traditional liquid electrolytes, SSEs has the advantages of low flammability, high thermal stability, no leakage, low explosion risk, and so on. More importantly, SSEs can effectively inhibit the growth of dendrite due to its high mechanical modulus, which makes the KMB system (K‐S, K‐O_2_ battery, etc.) achieve great development. Recently, several types of SSEs have been designed, including polymer electrolytes and ISEs for K‐based batteries.^[^ ^31^ ^]^

### 4.1 Polymer electrolytes

**FIGURE 17:**
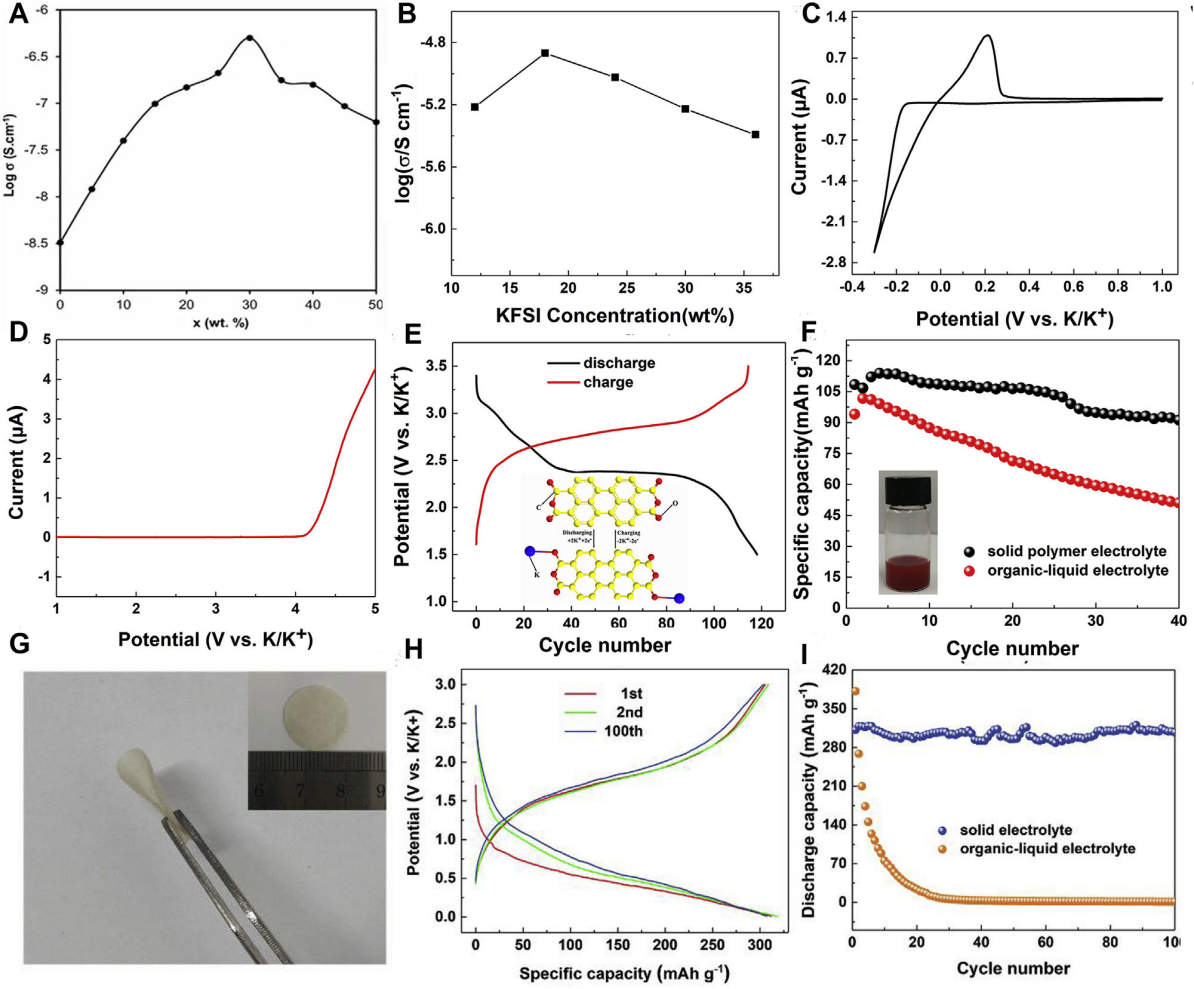
(A) ‘Log *σ*–x’ curve for the SPEs. Reproduced with permission.^[^ ^152^ ^]^ Copyright 2016, RSC. (B) Ionic conductivity of the Poly (propylene carbonate) (PPC/KFSI) electrolyte with increasing concentration of KFSI. (C) CV measurement of K metal ǁ stainless steel cell in the PPC/KFSI electrolyte. (D) The LSV curves for the PPC/KFSI electrolyte. (E) Charge/discharge curves of PTCDA with PPC/KFSI electrolyte at 10 mA g^−1^. The inset image is the schematic diagram for the reaction mechanism of PTCDA cathode in PIBs. (F) Cycle performance of PTCDA with different electrolytes at 20 mA g^−1^. The inset digital image shows the solubility of PTCDA. Reproduced with permission.^[^ ^154^ ^]^ Copyright 2018, Elsevier. (G) Images of flexible self‐supporting membrane. (H) Charge/discharge curves of PIBs with PEO/KFSI solid electrolyte at 25 mA g^−1^. (I) Cycle performance of Ni_3_S_2_@Ni electrode with PEO/KFSI and organic electrolyte at 25 mA g^−1^. Reproduced with permission.^[^ ^155^ ^]^ Copyright 2019, Elsevier

Polymer electrolytes (PEs) have emerged as promising materials for K‐based batteries due to its competitive advantages over liquid organic electrolytes and ISEs. On the one hand, compared with organic electrolytes, PEs with appropriate mechanical strength could effectively suppresses the growth of dendrites and accommodate volume changes of electrode materials. On the other hand, compared to ISEs, PEs emerged as advantageous materials for flexibility, easy film forming, excellent interface compatibility with electrodes.^[^ ^18^ ^]^ The research on K^+^ SPEs can be traced back to 1986 by Stevens group.^[^ ^151^ ^]^ The discovered PEO/KAg_4_I_5_ displayed ionic conductivity of 2 × 10^−3^ S cm^−1^ at room temperature with a low activation energy of 0.16 eV. Furthermore, PEO/KAg_4_I_5_ was certainly much more stable as a crystalline powder than pure KAg_4_I_5_. Then, Chandra. reported a SPE film [(1‐*x*) PEO/*x* KBr (0 < *x* < 50 wt%)], and found that ionic conductivity (*σ*) increased with the increasing of salt in the host polymer PEO.^[^ ^152^ ^]^ It displayed maximal *σ* (5.0 × 10^−7^) at 30 wt% of salt KBr (Figure 17A). Agrawal et al. synthesized 95PEO/5CH_3_COOK SPE films by hot‐presscast method, and it delivered a conductivity of 2.74 × 10^−7^ S cm^−1^ at RT.^[^ ^153^ ^]^

Unfortunately, the low ionic conductivity is unable to effectively facilitate K^+^ in potassium‐based batteries. Therefore, various polymer matrix materials with different K salts have been designed, such as, poly(vinyl alcohol), PVDF‐HFP, poly(vinylpyrrolidone,^[^ ^156^ ^]^ and PAN, etc. (Table 5). Feng et al. developed all‐solid‐state battery based on the PPC/KFSI electrolyte with 3,4,9,10‐perylene‐tetracarboxylicacid‐dianhydride (PTCDA) cathode for the first time. Figure 17B illustrates the PPC/KFSI showed maximal ionic conductivity of 1.36 × 10^−5^ S cm^−1^ (20°C) with 18 wt % KFSI. Moreover, the CV and LSV profiles revealed K metal could reversibly plate/stripe in a wide electrochemical window (Figure 17C,D). The all‐solid‐state battery delivered a high capacity of 118 mA h g^−1^ for the first cycle at 10 mA g^−1^, and a long cycle life (Figure 17E,F).^[^ ^154^ ^]^ Furthermore, the K||50PEO/50KFSI||Ni_3_S_2_@Ni full cell with the SPE (50PEO/50KFSI) (Figure 17G) displayed high capacity and stable cycle performance (Figure 17H,I).^[^ ^155^ ^]^ At present, the low ionic conductivity and mechanical strength, as well as, narrow working voltage for PEs, limit its practical application in K‐based battery.

**TABLE 5:** Property parameters of polymer electrolytes for PIBs

Classification	Electrolyte composition	Ionic conductivity (S cm^−1^)	Activation energies (eV)	Electrochemical stable voltage (V)
Gel polymer electrolyte	70PAN/30KI^[^ ^131^ ^]^	2.089 × 10^−5^	0.35	–
	85PAU/25KI^[^ ^157^ ^]^	1.59 × 10^−4^	–	–
	PMMA/KPF_6_^[^ ^132^ ^]^	3.7 × 10^−2^	2.5–4.9	0.27
Solid polymer electrolytes	PEO/KHCO_3_ (70/30)^[^ ^158^ ^]^	1.4 × 10^−7^	Region I, 0.21 Region II, 0.35	–
	PEO/KIO_3_ (73/30)^[^ ^159^ ^]^	4.4 × 10^−7^	0.17	–
	Sago starch/KI (70/30^[^ ^160^ ^]^	6.01 × 10^−6^		2 V
	PVP/KIO_4_ (85/15)^[^ ^161^ ^]^	1.421 × 10^−4^	0.21	–
	PVC/PEO/KCl (42.5/42.5/15)^[^ ^162^ ^]^	8.29 × 10^−6^	0.48	–
	PVC/PEO/KI (42.5/42.5/15)^[^ ^163^ ^]^	3.66 × 10^−4^	0.28	–
	PVA/CH_3_COOK (70/30)^[^ ^164^ ^]^	4.82 × 10^−7^	Region I, 0.7927 Region II, 0.1563	–
	95(70PEO/30KBr)+5SiO_2_^[^ ^159^ ^]^	2.5 × 10^−5^	0.34	–
	PPPCB/KFSI^[^ ^154^ ^]^	1.36 × 10^−5^	–	4.15 V
	95(80PEO/20KI)+ 5TiO_2_^[^ ^165^ ^]^	8.7 × 10^−5^	0.31	
	85PVA/15KCl^[^ ^166^ ^]^	9.68 × 10^−7^	0.29	
Inorganic solid electrolytes	K_2_Fe_4_O_7_	5 × 10^−2^	0.08 (a‐axis) 0.16 (c‐axis)	−2 to 5 V
	K_3_Sb_4_O_10_(BO_3_)^[^ ^167^ ^]^	1.5 × 10^−4^ (400°C)	0.325	–
	K_2_Mg_2_TeO_6_^[^ ^168^ ^]^	3.8 × 10^−2^ (300°C)	–	–
	K‐BASE	0.01 (150°C)	0.27	–

### 4.2 Inorganic solid electrolytes

**FIGURE 18:**
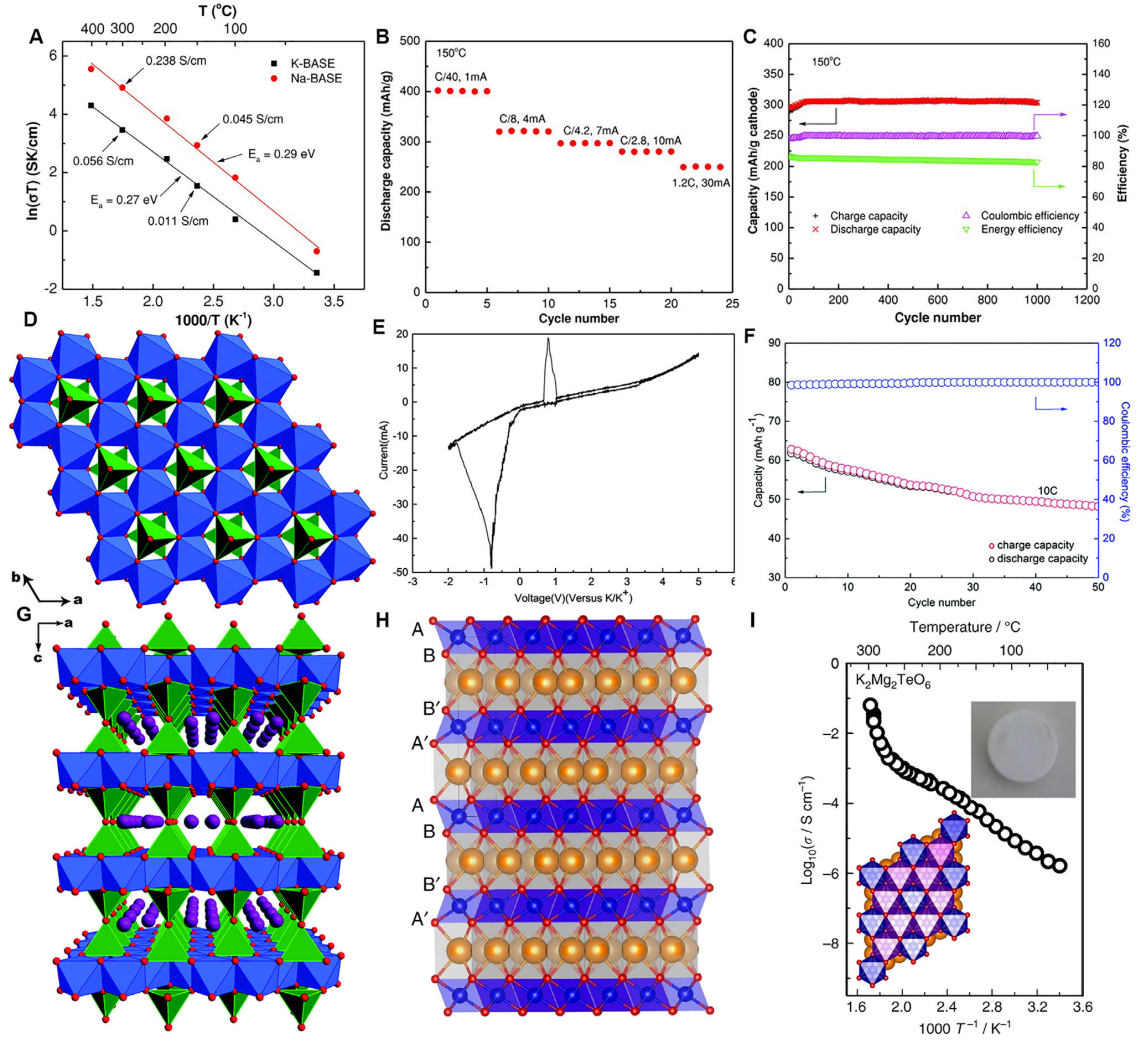
(A) Arrhenius plot of K‐ and Na‐BASE conductors. (B) Rate capability of a K‐S cell at 150°C (cell active area: 3 cm^2^). (C) Cycle performance and CEs of a K‐S cell. Reproduced with permission.^[^ ^172^ ^]^ Copyright 2015, Wiley. (D) 2D layer composed of FeO_6_ octahedra, and FeO_4_ tetrahedra with 1D 3‐ring channels. (E) CV measurement of the K/K_2_Fe_4_O_7_/Pt cell. (F) Cycle performance of K||K_2_Fe_4_O_7_||PBA battery at 10 C. (G) 3D open framework structure of K_2_Fe_4_O_7_ viewed along b‐axis. Reproduced with permission.^[^ ^175^ ^]^ Copyright 2018, RSC. (H) Crystal structure of K_2_Mg_2_TeO_6_ along *c*‐axis: Mg, Te, K, O are shown by purple, blue, brown red. (I) Arrhenius plot of K_2_Mg_2_TeO_6_. Reproduced with permission.^[^ ^168^ ^]^ Copyright 2018, Springer Nature

Compared with PEs, ISEs generally possess high ionic conductivities (>0.1 mS cm^−1^ at RT), high mechanical modulus (>1 GPa for oxides), wide excellent thermal/electrochemical stability windows (>4.0 V).^[^ ^169^ ^]^ In 1960s, β‐alumina solid electrolyte (BASE) with molten electrodes had been applied in Na‐S batteries by Yao and Kummer.^[^ ^170^ ^]^ Subsequently, a serious of compounds of M_2_O⋅*x*Al_2_O_3_ (where, M = Na^+^, K^+^, Rb^+^, Ag^+^, 5 < *x* < 11) by ion exchange method were developed.^[^ ^171^ ^]^ In 2015, Liu et al. first assembled K‐BASE for K‐S battery, which could be operated at moderate temperatures (150°C), much lower than of Na‐S battery.^[^ ^172^ ^]^ The conductivity of K‐BASE was ≈0.056 at 300°C (Figure 18A). The K‐S batteries showed excellent rate performance with retention rate of 75% at 1.2 C compared to the rate of C/40 (Figure 18B). In addition, the K‐S battery displayed stable cycle performance over 1000 cycles at C/4.2 (Figure 18C).

In addition, various ISEs with suitable 1D, 2D, or 3D channels, where K^+^ charge carrier ions could move quickly along, have been studied. K_3_Sb_4_O_10_(BO_3_) compound with 1D K^+^ ionic channel along the a‐b directions was prepared (Figure 18D).^[^ ^173^ ^]^ It showed about 1.5 × 10^−4^ S cm^−1^ at 400°C with an activation energy of 0.325 eV. Delmas et al. explored new lamellar structures of general formula KLO_2_‐MO_2_, where L = In or Sc, and M = Zr, Sn, or Pb. Above 250°C, the conductivity of K_0.72_1n_0.72_Hf_0.28_O_2_ exceeded that of sintered β‐alumina, although the activation energy was greater.^[^ ^174^ ^]^ Recently, an open‐framework potassium ferrite K_2_Fe_4_O_7_ compound with 2D 6‐ring channels paralleling to the a‐b planes and smaller 1D 3‐ring channels at the c‐axis was prepared by hydrothermal method, as illustrated in Figure 18D,G.^[^ ^175^ ^]^ Notably, K_2_Fe_4_O_7_ achieved high ionic conductivity (5.0 × 10^−2^ S cm^−1^ at RT) and wide working voltage (5 V vs. K/K^+^), which mainly derived from the 2D channel with lower migration resistance (Figure 18E). The all‐solid‐state K metal battery with PBA as cathode, and K_2_Fe_4_O_7_ as the electrolyte, could deliver stable cycle performance over 50 cycles with retention rate of 78% (Figure 18F). In addition, Tellurates compounds K_2_M_2_TeO_6_ (M  =  transition metal) with layered honeycomb frameworks was prepared, in which K vacancy resulted in fast K^+^ migration along the layers (Figure 18H).^[^ ^168^ ^]^ Among tellurates, K_2_Mg_2_TeO_6_ exhibited highest ionic conductivity of 40 mS cm^−1^ at 300°C (Figure 18I).

NASICON (Na_l+_ *_x_* Zr_2_P_3‐_ *_x_* Si*_x_* O_12_, 0 < *x* < 3) structure was built by (Si, P)O_4_ tetrahedra and ZrO_6_ octahedra, and it provided a 3D open network for Na^+^.^[^ ^176^ ^]^ Na_3_Zr_2_P_1_Si_2_O_12_ had a Na^+^‐ion conductivity at 300°C of 0.2 S cm^−1^, comparable to β‐alumina. For the development of potassium ion conductors, it is of interest to prepare NASICON‐like compounds with potassium. K_2_YZr(PO_4_)_3_ was reported, in which the K^+^ ions were situated in statistical distribution of [Y [6] Zr [6] (PO_4_)_3_]^2−^ units, and it showed ion conductivity of 1.19 × 10^− 3^ S cm^−1^ at 800°C.^[^ ^177^ ^]^

Moreover, theoretical calculations have applied to predict potential K^+^ ionic conductors. For example, Rao et al. employed the bond valence method to identify new fast ionic conductor.^[^ ^178^ ^]^ Examination of the crystal structures revealed that the highest accessible values in Li^+^, Na^+^, and K^+^ oxides most frequently occurred in Li_3_ *_x_* La_2/3−_ *_x_* TiO_3_ perovskite type, P2‐ and β‐NaFeO_2_ types, and birnessite type, respectively. Furthermore, the high‐throughput geometrical‐topological approach and precise DFT modeling have been applied for new promising solid electrolytes.^[^ ^179^ ^]^ It was concluded that compounds K_5_As_3_O_10_, K_2_Zn_3_O_4_, K_2_Sb_4_O_11_, K_4_V_2_O_7_, K_3_NbP_2_O_9_, K_3_NbAs_2_O_9_, and K_6_CuSi_2_O_8_ were prospective as ionic conductors. In particular, K_2_Al_2_Sb_2_O_7_ and K_4_V_2_O_7_ were the most competitive 2D and 3D ion conductors, respectively. Recently, Xiao et al. also anticipated a novel K^+^ conductor Al‐doped K_2_CdO_2_ (2.2 × 10^−5^ S cm^−1^ at 27°C) by high‐throughput computational method.^[^ ^180^ ^]^ For ISEs, the main shortages of K^+^‐based ISEs are poor interface instability and compatibility with electrodes. What is worse, the ISEs researches are still in its infancy, only a few K^+^ ionic conductors were developed (Table 5).

## 5 CONCLUSIONS AND OUTLOOK

The low cost and abundance of K makes potassium‐based batteries promising for large‐scale ESSs. The lithium ion batteries have reached the threshold for application, whereas the researches on potassium‐based batteries are still in its infancy, especially for electrolytes. Herein, we exhaustively summarize the design principles and recent development of K^+^ electrolytes, meanwhile, offer the potential directions on the unresolved issues.

### 5.1 Developing novel electrolytes and characterization techniques

The electrolyte should be given priority for high‐performance K‐based batteries. However, most literature has focused on a limited range of organic electrolytes based on KPF_6_ and KFSI, and these have not resulted in theoretical electrode performance. A wider variety of electrolyte formulations should be investigated, such as, additives, novel solvents, salts, optimizing concentration of salts. Simultaneously, it is important to understand the fundamental interactions in K^+^ electrolytes at the atomic level, the thermodynamics and kinetics of electrolyte reduction reactions, and SEI formation, applied by Li bond theory, high‐throughput screening and machine learning methods. Furthermore, the computational modeling can be integrated with complement each other to battery performance for the development of a highly efficient battery in the future. Similar to lithium‐based batteries, it is necessary to develop alternative all solid‐state‐based electrolytes to avoid excess side reactions. Currently, the all solid‐state ionic conductors with high conductivity at room temperature are core problems. Furthermore, computational chemistry could provide a potential approach to predict and design new solid‐state electrolytes based on materials databases.

The dynamic features of electrolyte and failure products may provide a possible way to explore substituted electrolyte. However, standard measurement techniques for the interface cannot be used for K‐ion due to its high reactivity. In this regard, all kinds of in situ/operando characterization techniques worked under real battery conditions, are necessary. Furthermore, the deep understanding on the formation and regulating of K^+^ solvation structure, and the mechanisms of K^+^ desolvation on different electrode surface will enable development of long cycle life of PIBs.

### 5.2 Improve interfacial stability

The electrolytes and interphases are inseparable, and closely interweave by electro/chemical reactions. As an indispensable part, the interphases on cathode and anode play a vital role in K based batteries, including the initial capacity fading, low coulombic efficiency, short cycle life, and safety. Therefore, the understanding of SEI formation, and dynamic change over cycles help solve the challenge of dendrite formation. For liquid organic electrolyte, a handful of solvents and salts including ethers, nonflammable phosphate, KFSI, etc., have emerged as promising for their ability to form stable inorganic SEI on the anodes. A drawback is that these components typically exhibit low coulombic efficiency and short cycle life. The LHCEs and perfluorinated electrolytes have been demonstrated as effective ways to improve the cycling performance, but highly fluorinated solvents and salts are deficient. Optimizing the electrolyte formulation, and artificial SEI, are effective ways to solve the challenge of SEI instability. For solid‐state electrolyte, the poor interface compatibility and ionic conductivity should be give given priority. Understanding the mechanism of formation, and dynamic features of the interphases are the guide for designing stable layers.

### 5.3 Developing stable K metal anode or alternatives

For K metal anode, new issues are introduced, dendrites growth, cross‐talk of intermediates, and worsening side. These shortcomings make potassium metal difficult to meet the requirements of reference electrodes, which exhibits a stable rest potential, is chemically inert for reliable electrochemical measurements. In K metal batteries, these disadvantages create an inaccurate evaluation of electrode materials, severely affecting both rate and cycle life. Despite of the similarities to Li/Na metals, the differences in their energy chemistries inevitably affect their electrochemical processes and their practical applications as working anodes. Therefore, more research should be focus on K metal or alternatives.

### 5.4 Full‐cell design

To reach the practical application goal, full‐cell fabrication is necessary. To realize practical applications of potassium‐based batteries at room temperature, we suggest that further studies focus on the following aspects. First, improving coulombic efficiency by designing electrolytes. Second, optimizing the comprehensive performance on the electrodes/electrolytes interface through experimental studies and advanced in situ techniques (such as, XRD, XPS, and TEM). Finally, realizing large‐scale production of high‐performance K‐based batteries via developing low‐cost electrodes, and binders and separators.

## CONFLICTS OF INTEREST

There are no conflicts to declare.

## References

[1] X. Zeng , M. Li , D. A. El‐Hady , W. Alshitari , A. S. Al‐Bogami , J. Lu , K. Amine , Adv. Energy Mater. 2019, 9, 1900161.

[2] W. Zhang , Y. Liu , Z. Guo , Sci. Adv. 2019, 5, 7412. 10.1126/sciadv.aav7412 PMC6510555 31093528

[3] a) P. Liu , Y. Wang , H. Hao , S. Basu , X. Feng , Y. Xu , J. A. Boscoboinik , J. Nanda , J. Watt , D. Mitlin , Adv. Mater. 2020, 32, 2002908; 10.1002/adma.202002908 33135265

[4] N. Matsuura , K. Umemoto , Z. i. Takeuchi , Bull. Chem. Soc. Jpn. 1974, 47, 813.

[5] Y. Matsuda , H. Nakashima , M. Morita , Y. Takasu , J. Electrochem. Soc. 1981, 128, 2552.

[6] T. A. Pham , K. E. Kweon , A. Samanta , V. Lordi , J. E. Pask , J. Phys. Chem. C 2017, 121, 21913.

[7] a) M. Okoshi , Y. Yamada , S. Komaba , A. Yamada , H. Nakai , J. Electrochem. Soc. 2017, 164, A54;

[8] Y. Li , Y. Lu , P. Adelhelm , M. M. Titirici , Y. Hu , Chem. Soc. Rev. 2019, 48, 4655. 31294739 10.1039/c9cs00162j

[9] a) Q. Gan , J. Xie , Y. Zhu , F. Zhang , P. Zhang , Z. He , S. Liu , ACS Appl. Mater. Interfaces 2019, 11, 930; 30550259 10.1021/acsami.8b18553

[10] a) H. Li , C. Zhao , Y. Yin , Y. Zou , Y. Xia , Q. An , Z. Jian , W. Chen , Nanoscale 2020, 12, 4309; 32026906 10.1039/c9nr09867d

[11] a) S. Xu , Y. Ding , X. Liu , Q. Zhang , K. Wang , J. Chen , Adv. Energy Mater. 2018, 8, 1802175;

[12] a) L. Xue , Y. Li , H. Gao , W. Zhou , X. Lü , W. Kaveevivitchai , A. Manthiram , J. B. Goodenough , J. Am. Chem. Soc. 2017, 139, 2164; 28125230 10.1021/jacs.6b12598

[13] H. Kim , D. H. Seo , A. Urban , J. Lee , D. H. Kwon , S. H. Bo , T. Shi , J. K. Papp , B. D. McCloskey , G. Ceder , Chem. Mater. 2018, 30, 6532.

[14] a) Y. Hu , W. Tang , Q. Yu , X. Wang , W. Liu , J. Hu , C. Fan , Adv. Funct. Mater. 2020, 30, 2000675;

[15] K. Xu , Chem. Rev. 2004, 104, 4303. 15669157 10.1021/cr030203g

[16] M. Moshkovich , Y. Gofer , D. Aurbach , J. Electrochem. Soc. 2001, 148, E155.

[17] L. Ni , M. Osenberg , H. Liu , A. Hilger , L. Chen , D. Zhou , K. Dong , T. Arlt , X. Yao , X. Wang , Nano Energy 2021, 83, 105841.

[18] K. Xu , Chem. Rev. 2014, 114, 11503. 25351820 10.1021/cr500003w

[19] S. Alipoori , S. Mazinani , S. H. Aboutalebi , F. Sharif , J. Energy Storage 2020, 27, 101072.

[20] E. Kim , J. Han , S. Ryu , Y. Choi , J. Yoo , Materials 2021, 14, 4000. 34300918 10.3390/ma14144000 PMC8308040

[21] S. Amara , J. Toulc'Hoat , L. Timperman , A. Biller , H. Galiano , C. Marcel , M. Ledigabel , M. Anouti , ChemPhysChem 2019, 20, 581. 30614615 10.1002/cphc.201801064

[22] M. Okoshi , Y. Yamada , S. Komaba , A. Yamada , H. Nakai , J. Electrochem. Soc. 2017, 164, A54.

[23] T. Hosaka , K. Kubota , H. Kojima , S. Komaba , Chem. Commun. 2018, 54, 8387. 10.1039/c8cc04433c 29998275

[24] R. Hagiwara , K. Tamaki , K. Kubota , T. Goto , T. Nohira , J. Chem. Eng. Data 2008, 53, 355.

[25] K. Kubota , M. Dahbi , T. Hosaka , S. Kumakura , S. Komaba , Chem. Rec. 2018, 18, 459. 29442429 10.1002/tcr.201700057

[26] Y. Hu , Y. Lu , ACS Energy Lett. 2020, 5, 3633.

[27] K. M. Diederichsen , E. J. McShane , B. D. McCloskey , ACS Energy Lett. 2017, 2, 2563.

[28] S. Zugmann , M. Fleischmann , M. Amereller , R. M. Gschwind , H. D. Wiemhöfer , H. J. Gores , Electrochim. Acta 2011, 56, 3926.

[29] a) K. Mukai , T. Inoue , Y. Kato , S. Shirai , ACS Omega 2017, 2, 864; 31457478 10.1021/acsomega.6b00551 PMC6640915

[30] X. Chen , X. Shen , B. Li , H. J. Peng , X. B. Cheng , B. Q. Li , X. Q. Zhang , J. Q. Huang , Q. Zhang , Angew. Chem. Int. Ed. 2018, 57, 734. 10.1002/anie.201711552 29178154

[31] H. Yin , C. Han , Q. Liu , F. Wu , F. Zhang , Y. Tang , Small 2021, 17, 2006627. 10.1002/smll.202006627 34047049

[32] X. Q. Zhang , X. Chen , X. B. Cheng , B. Q. Li , X. Shen , C. Yan , J. Q. Huang , Q. Zhang , Angew. Chem. Int. Ed. 2018, 130, 5399.

[33] P. Peljo , H. H. Girault , Energy Environ. Sci. 2018, 11, 2306. 10.1039/d4ee04560b PMC11753199 39866363

[34] X. Xu , K. Lin , D. Zhou , Q. Liu , X. Qin , S. Wang , S. He , F. Kang , B. Li , G. Wang , Chem 2020, 6, 902.

[35] a) X. Chen , H. Li , X. Shen , Q. Zhang , Angew. Chem. Int. Ed. 2018, 57, 16643; 10.1002/anie.201809203 30334312

[36] a) Z. Chen , Y. Tang , X. Du , B. Chen , G. Lu , X. Han , Y. Zhang , W. Yang , P. Han , J. Zhao , Angew. Chem. Int. Ed. 2020, 59, 21769; 10.1002/anie.202010423 32812326

[37] S. K. Heiskanen , J. Kim , B. L. Lucht , Joule 2019, 3, 2322.

[38] R. Mogensen , D. Brandell , R. Younesi , ACS Energy Lett. 2016, 1, 1173.

[39] T. Hosaka , K. Kubota , A. S. Hameed , S. Komaba , Chem. Rev. 2020, 120, 6358. 31939297 10.1021/acs.chemrev.9b00463

[40] Y. Gu , W. Wang , Y. Li , Q. Wu , S. Tang , J. Yan , M. Zheng , D. Wu , C. Fan , W. Hu , Z.‐B. Chen , Y. Fang , Q.‐H. Zhang , Q.‐F. Dong , B.‐W. Mao , Nat. Commun. 2018, 9, 1339. 29632301 10.1038/s41467-018-03466-8 PMC5890267

[41] a) N. Yabuuchi , K. Kubota , M. Dahbi , S. Komaba , Chem. Rev. 2014, 114, 11636; 25390643 10.1021/cr500192f

[42] M. Hess , Electrochim. Acta. 2017, 244, 69.

[43] Y. Qi , J. Li , W. Zhong , S. Bao , M. Xu , Chem. Eng. J. 2021, 417, 128159.

[44] L. Zhu , Z. Zhang , J. Luo , H. Zhang , Y. Qu , Z. Yang , Carbon 2021, 174, 317.

[45] Y. Jiang , Y. Yang , R. Xu , X. Cheng , H. Huang , P. Shi , Y. Yao , H. Yang , D. Li , X. Zhou , ACS Nano 2021, 15, 10217. 34037375 10.1021/acsnano.1c02275

[46] D. Su , Y. Pei , L. Liu , Z. Liu , J. Liu , M. Yang , J. Wen , J. Dai , H. Deng , G. Cao , Nano‐Micro Lett. 2021, 13, 107. 10.1007/s40820-021-00632-4 PMC8035377 34138372

[47] J. Cao , L. Wang , D. Li , Z. Yuan , H. Xu , J. Li , R. Chen , V. Shulga , G. Shen , W. Han , Adv. Mater. 2021, 33, 2101535. 10.1002/adma.202101535 34288161

[48] D. Zhou , J. Yi , X. Zhao , J. Yang , H. Lu , L.‐Z. Fan , Chem. Eng. J. 2021, 413, 127508.

[49] L. Zhou , Z. Cao , J. Zhang , H. Cheng , G. Liu , G. T. Park , L. Cavallo , L. Wang , H. N. Alshareef , Y. K. Sun , Adv. Mater. 2021, 33, 2005993. 10.1002/adma.202005993 33470482

[50] S. Haghighat‐Shishavan , M. Nazarian‐Samani , M. Nazarian‐Samani , K.‐B. Kim , Energy Storage Mater. 2021, 39, 96.

[51] Q. Pan , Y. Zheng , Z. Tong , L. Shi , Y. Tang , Angew. Chem. Int. Ed. 2021, 60, 11835. 10.1002/anie.202103052 33723907

[52] L. Liu , J. Liang , W. Wang , C. Han , Q. Xia , X. Ke , J. Liu , Q. Gu , Z. Shi , S. Chou , ACS Appl. Mater. Interfaces 2021, 13, 28369. 34107212 10.1021/acsami.1c07220

[53] X. Niu , J. Qu , Y. Hong , L. Deng , R. Wang , M. Feng , J. Wang , L. Zeng , Q. Zhang , L. Guo , J. Mater. Chem. A 2021, 9, 13125.

[54] L. Deng , J. Qu , X. Niu , J. Liu , J. Zhang , Y. Hong , M. Feng , J. Wang , M. Hu , L. Zeng , Q. Zhang , L. Guo , Y. Zhu , Nat. Commun. 2021, 12, 2167. 33846311 10.1038/s41467-021-22499-0 PMC8041879

[55] H. Park , W. Ko , Y. Lee , J. Kang , J. Ahn , J.‐K. Yoo , J. Kim , J. Mater. Chem. A 2021, 9, 11802.

[56] J. Kang , H. Park , W. Ko , Y. Lee , J. Ahn , J.‐K. Yoo , S. H. Song , H. Kim , J. Kim , J. Mater. Chem. A 2021, 9, 9898.

[57] S. Liu , J. Mao , L. Zhang , W. K. Pang , A. Du , Z. Guo , Adv. Mater. 2021, 33, 2006313. 10.1002/adma.202006313 33225551

[58] J. Zhao , X. Zou , Y. Zhu , Y. Xu , C. Wang , Adv. Funct. Mater. 2016, 26, 8103.

[59] a) A. Ponrouch , E. Marchante , M. Courty , J. M. Tarascon , M. R. Palacin , Energy Environ. Sci. 2012, 5, 8572;

[60] H. Wang , D. Yu , X. Wang , Z. Niu , M. Chen , L. Cheng , W. Zhou , L. Guo , Angew. Chem. Int. Ed. 2019, 131, 16603. 10.1002/anie.201908607 31482655

[61] J. Li , N. Zhuang , J. Xie , X. Li , W. Zhuo , H. Wang , J. B. Na , X. Li , Y. Yamauchi , W. Mai , Adv. Energy Mater. 2020, 10, 1903455.

[62] S. Liu , J. Mao , Q. Zhang , Z. Wang , W. K. Pang , L. Zhang , A. Du , V. Sencadas , W. Zhang , Z. Guo , Angew. Chem. Int. Ed. 2020, 59, 3638. 10.1002/anie.201913174 31840345

[63] J. Liao , Q. Hu , Y. Yu , H. Wang , Z. Tang , Z. Wen , C. Chen , J. Mater. Chem. A 2017, 5, 19017.

[64] M. Gu , L. Fan , J. Zhou , A. M. Rao , B. Lu , ACS Nano 2021, 15, 9167. 33938743 10.1021/acsnano.1c02727

[65] Q. Zhang , J. Mao , W. K. Pang , T. Zheng , V. Sencadas , Y. Chen , Y. Liu , Z. Guo , Adv. Energy Mater. 2018, 8, 1703288.

[66] T. Hosaka , S. Muratsubaki , K. Kubota , H. Onuma , S. Komaba , J. Phys. Chem. Lett. 2019, 10, 3296. 31042388 10.1021/acs.jpclett.9b00711

[67] L. Fan , S. Chen , R. Ma , J. Wang , L. Wang , Q. Zhang , E. Zhang , Z. Liu , B. Lu , Small 2018, 14, 1801806. 10.1002/smll.201801806 29956476

[68] J. Touja , P. N. Le Pham , N. Louvain , L. Monconduit , L. Stievano , Chem. Commun. 2020, 56, 14673. 10.1039/d0cc05024e 33159783

[69] A. M. Haregewoin , A. S. Wotango , B.‐J. Hwang , Energy Environ. Sci. 2016, 9, 1955.

[70] J. Fondard , E. Irisarri , C. Courreges , M. R. Palacín , A. Ponrouch , R. Dedryvère , J. Electrochem. Soc. 2020, 167, 070526.

[71] N. S. Choi , K. H. Yew , K. Y. Lee , M. Sung , H. Kim , S. S. Kim , J. Power Sources 2006, 161, 1254.

[72] A. K. Haridas , Q. A. Nguyen , T. Terlier , R. Blaser , S. L. Biswal , ACS Appl. Mater. Interfaces 2021, 13, 2662. 33423465 10.1021/acsami.0c19347

[73] X. Bie , K. Kubota , T. Hosaka , K. Chihara , S. Komaba , J. Mater. Chem. A 2017, 5, 4325.

[74] G. He , L. F. Nazar , ACS Energy Lett. 2017, 2, 1122.

[75] W. Zhang , W. K. Pang , V. Sencadas , Z. Guo , Joule 2018, 2, 1534.

[76] W. Zhang , Z. Wu , J. Zhang , G. Liu , N.‐H. Yang , R.‐S. Liu , W. K. Pang , W. Li , Z. Guo , Nano Energy 2018, 53, 967.

[77] G. Liu , Z. Cao , L. Zhou , J. Zhang , Q. Sun , J. Y. Hwang , L. Cavallo , L. Wang , Y. K. Sun , J. Ming , Adv. Funct. Mater. 2020, 30, 2001934.

[78] a) H. Yang , C. Y. Chen , J. Hwang , K. Kubota , K. Matsumoto , R. Hagiwara , ACS Appl. Mater. Interfaces 2020, 12, 36168; 32692540 10.1021/acsami.0c09562

[79] D. Liu , Z. Yang , W. Li , S. Qiu , Y. Luo , Electrochim. Acta 2010, 55, 1013.

[80] a) Y. Liu , F. Fan , J. Wang , Y. Liu , H. Chen , K. L. Jungjohann , Y. Xu , Y. Zhu , D. Bigio , T. Zhu , Nano Lett. 2014, 14, 3445; 24823874 10.1021/nl500970a

[81] a) S. Komaba , T. Hasegawa , M. Dahbi , K. Kubota , Electrochem. Commun. 2015, 60, 172;

[82] J. Y. Hwang , S. T. Myung , Y. K. Sun , Adv. Funct. Mater. 2018, 28, 1802938.

[83] B. Huang , Y. Liu , Z. Lu , M. Shen , J. Zhou , J. Ren , X. Li , S. Liao , ACS Sustainable Chem. Eng. 2019, 7, 16659.

[84] C. Zhang , Y. Xu , M. Zhou , L. Liang , H. Dong , M. Wu , Y. Yang , Y. Lei , Adv. Funct. Mater. 2017, 27, 1604307.

[85] Y. Luo , B. Shen , B. Guo , L. Hu , Q. Xu , R. Zhan , Y. Zhang , S. Bao , M. Xu , J. Phys. Chem. Solids 2018, 122, 31.

[86] B. Huang , Y. Shao , Y. Liu , Z. Lu , X. Lu , S. Liao , ACS Appl. Energy Mater. 2019, 2, 6528.

[87] Q. Xue , L. Li , Y. Huang , R. Huang , F. Wu , R. Chen , ACS Appl. Mater. Interfaces 2019, 11, 22339. 31149796 10.1021/acsami.9b04579

[88] Y. Zhu , Q. Zhang , X. Yang , E. Zhao , T. Sun , X. Zhang , S. Wang , X. Yu , J. Yan , Q. Jiang , Chem 2019, 5, 168.

[89] C. Vaalma , G. A. Giffin , D. Buchholz , S. Passerini , J. Electrochem. Soc. 2016, 163, A1295.

[90] J. U. Choi , Y. J. Park , J. H. Jo , Y. H. Jung , D. C. Ahn , T. Y. Jeon , K. S. Lee , H. Kim , S. Lee , J. Kim , Energy Storage Mater. 2020, 27, 342.

[91] J. U. Choi , J. Kim , J. Y. Hwang , J. H. Jo , Y. K. Sun , S. T. Myung , Nano Energy 2019, 61, 284.

[92] X. Zhang , Y. Yang , X. Qu , Z. Wei , G. Sun , K. Zheng , H. Yu , F. Du , Adv. Funct. Mater. 2019, 29, 1905679.

[93] Y. Hironaka , K. Kubota , S. Komaba , Chem. Commun. 2017, 53, 3693. 10.1039/c7cc00806f 28294241

[94] J. Han , G. Li , F. Liu , M. Wang , Y. Zhang , L. Hu , C. Dai , M. Xu , Chem. Commun. 2017, 53, 1805. 10.1039/c6cc10065a 28111676

[95] S. Zheng , S. Cheng , S. Xiao , L. Hu , Z. Chen , B. Huang , Q. Liu , J. Yang , Q. Chen , J. Alloys Compd. 2020, 815, 152379.

[96] X. Lin , J. Huang , H. Tan , J. Huang , B. Zhang , Energy Storage Mater. 2019, 16, 97.

[97] J. Liao , Q. Hu , B. Che , X. Ding , F. Chen , C. Chen , J. Mater. Chem. A 2019, 7, 15244.

[98] H. Park , H. Kim , W. Ko , J. H. Jo , Y. Lee , J. Kang , I. Park , S. T. Myung , J. Kim , Energy Storage Mater. 2020, 28, 47.

[99] N. Voronina , J. H. Jo , A. Konarov , J. Kim , S. T. Myung , Small 2020, 16, 2001090. 10.1002/smll.202001090 32329570

[100] B. Ji , W. Yao , Y. Zheng , P. Kidkhunthod , X. Zhou , S. Tunmee , S. Sattayaporn , H. Cheng , H. He , Y. Tang , Nat. Commun. 2020, 11, 1225. 32144250 10.1038/s41467-020-15044-y PMC7060185

[101] Y. Chen , W. Luo , M. Carter , L. Zhou , J. Dai , K. Fu , S. Lacey , T. Li , J. Wan , X. Han , Nano Energy 2015, 18, 205.

[102] Z. Jian , Y. Liang , I. A. Rodríguez‐Pérez , Y. Yao , X. Ji , Electrochem. Commun. 2016, 71, 5.

[103] L. Fan , Q. Liu , Z. Xu , B. Lu , ACS Energy Lett. 2017, 2, 1614.

[104] Z. Tai , Q. Zhang , Y. Liu , H. Liu , S. Dou , Carbon 2017, 123, 54.

[105] Y. Zhao , L. Yang , C. Ma , G. Han , Energy Fuels 2020, 34, 8993.

[106] S. Wu , J. Mo , Y. Zeng , Y. Wang , A. Rawal , J. Scott , Z. Su , W. Ren , S. Chen , K. Wang , Small 2020, 16, 1903397. 10.1002/smll.201903397 31496028

[107] J. Li , Y. Li , X. Ma , K. Zhang , J. Hu , C. Yang , M. Liu , Chem. Eng. J. 2020, 384, 123328.

[108] R. Zhang , J. Bao , Y. Wang , C. Sun , Chem. Sci. 2018, 9, 6193. 30090306 10.1039/c8sc01848k PMC6062896

[109] G. W. Lee , B. H. Park , M. Nazarian‐Samani , Y. H. Kim , K. C. Roh , K. B. Kim , ACS Omega 2019, 4, 5304. 31459701 10.1021/acsomega.9b00045 PMC6648113

[110] X. Shi , L. Qin , G. Xu , S. Guo , S. Ma , Y. Zhao , J. Zhou , S. Liang , Chem. Commun. 2020, 56, 3713. 10.1039/d0cc01009j 32191784

[111] S. Lu , H. Wu , S. Xu , Y. Wang , J. Zhao , Y. Li , A. M. Abdelkader , J. Li , W. Wang , K. Xi , Small 2021, 17, 2005745. 10.1002/smll.202005745 33522048

[112] J. Chu , W. Wang , Q. Yu , C. Lao , L. Zhang , K. Xi , K. Han , L. Xing , L. Song , M. Wang , J. Mater. Chem. A 2020, 8, 779.

[113] I. Sultana , T. Ramireddy , M. M. Rahman , Y. Chen , A. M. Glushenkov , Chem. Commun. 2016, 52, 9279. 10.1039/c6cc03649j 27358087

[114] Y. Han , T. Li , Y. Li , J. Tian , Z. Yi , N. Lin , Y. Qian , Energy Storage Mater. 2019, 20, 46.

[115] P. Xiong , J. Wu , M. Zhou , Y. Xu , ACS Nano 2019, 14, 1018. 31860268 10.1021/acsnano.9b08526

[116] Y. Zhao , X. Ren , Z. Xing , D. Zhu , W. Tian , C. Guan , Y. Yang , W. Qin , J. Wang , L. Zhang , Small 2020, 16, 1905789. 10.1002/smll.201905789 31825563

[117] L. Li , L. Liu , Z. Hu , Y. Lu , Q. Liu , S. Jin , Q. Zhang , S. Zhao , S. L. Chou , Angew. Chem. Int. Ed. 2020, 132, 13017. 10.1002/anie.202001966 32298024

[118] L. Fan , R. Ma , Q. Zhang , X. Jia , B. Lu , Angew. Chem. Int. Ed. 2019, 131, 10610. 10.1002/anie.201904258 31162778

[119] a) L. Wang , J. Yang , J. Li , T. Chen , S. Chen , Z. Wu , J. Qiu , B. Wang , P. Gao , X. Niu , J. Power Sources 2019, 409, 24;

[120] L. Deng , T. Wang , Y. Hong , M. Feng , R. Wang , J. Zhang , Q. Zhang , J. Wang , L. Zeng , Y. Zhu , ACS Energy Lett. 2020, 5, 1916.

[121] T. Yamamoto , K. Matsumoto , R. Hagiwara , T. Nohira , J. Phys. Chem. C 2017, 121, 18450.

[122] H. Yamamoto , C. Y. Chen , K. Kubota , K. Matsumoto , R. Hagiwara , J. Phys. Chem. B 2020, 124, 6341. 32598152 10.1021/acs.jpcb.0c03272

[123] L. Samain , F. Grandjean , G. J. Long , P. Martinetto , P. Bordet , D. Strivay , J. Phys. Chem. C 2013, 117, 9693. 10.1107/S0909049513004585 23592626

[124] A. Eftekhari , J. Power Sources 2004, 126, 221.

[125] L. Xue , Y. Li , H. Gao , W. Zhou , X. Lü , W. Kaveevivitchai , A. Manthiram , J. B. Goodenough , J. Am. Chem. Soc. 2017, 139, 2164. 28125230 10.1021/jacs.6b12598

[126] N. Xiao , W. D. McCulloch , Y. Wu , J. Am. Chem. Soc. 2017, 139, 9475. 28662577 10.1021/jacs.7b04945

[127] H. Sun , P. Liang , G. Zhu , W. Hung , Y. Li , H. Tai , C. Huang , J. Li , Y. Meng , M. Angell , Proc. Natl. Acad. Sci. U. S. A. 2020, 117, 27847. 33106405 10.1073/pnas.2012716117 PMC7668001

[128] H. Liu , X. Cheng , Z. Jin , R. Zhang , G. Wang , L. Chen , Q. Liu , J. Huang , Q. Zhang , EnergyChem 2019, 1, 100003.

[129] a) P. Liu , D. Mitlin , Acc. Chem. Res. 2020, 53, 1161; 32466644 10.1021/acs.accounts.0c00099

[130] Y. Gu , W. W. Wang , Y. J. Li , Q. H. Wu , S. Tang , J. W. Yan , M. S. Zheng , D. Y. Wu , C. H. Fan , W. Q. Hu , Z. B. Chen , Y. Fang , Q. H. Zhang , Q. F. Dong , B. W. Mao , Nat. Commun. 2018, 2018, 5971646. 10.1038/s41467-018-03466-8 PMC5890267 29632301

[131] N. K. Jyothi , K. Venkataratnam , P. N. Murty , K. V. Kumar , Bull. Mater. Sci. 2016, 39, 1047.

[132] H. Gao , L. Xue , S. Xin , J. B. Goodenough , Angew. Chem. Int. Ed. 2018, 130, 5547. 10.1002/anie.201802248 29534324

[133] J. Lang , J. Li , X. Ou , F. Zhang , K. Shin , Y. Tang , ACS Appl. Mater. Interfaces 2019, 12, 2424. 31815432 10.1021/acsami.9b17635

[134] a) L. Qin , Y. Lei , H. Wang , J. Dong , Y. Wu , D. Zhai , F. Kang , Y. Tao , Q. H. Yang , Adv. Energy Mater. 2019, 9, 1901427;

[135] a) Q. Zhao , Y. Hu , K. Zhang , J. Chen , Inorg. Chem. 2014, 53, 9000; 25119141 10.1021/ic500919e

[136] M. Salama , Rosy , R. Attias , R. Yemini , Y. Gofer , D. Aurbach , M. Noked , ACS Energy Lett. 2019, 4, 436.

[137] Y. Yao , R. Xu , M. Chen , X. Cheng , S. Zeng , D. Li , X. Zhou , X. Wu , Y. Yu , ACS Nano 2019, 13, 4695. 30946566 10.1021/acsnano.9b00980

[138] X. Yu , A. Manthiram , Energy Storage Mater. 2018, 15, 368.

[139] L. Wang , J. Bao , Q. Liu , C.‐F. Sun , Energy Storage Mater. 2019, 18, 470.

[140] a) J. Zhu , J. Zou , H. Cheng , Y. Gu , Z. Lu , Green Energy Environ. 2019, 4, 345;

[141] Y. Liu , Z. Tai , Q. Zhang , H. Wang , W. K. Pang , H. K. Liu , K. Konstantinov , Z. Guo , Nano Energy 2017, 35, 36.

[142] R. Xu , Y. Yao , H. Wang , Y. Yuan , J. Wang , H. Yang , Y. Jiang , P. Shi , X. Wu , Z. Peng , Adv. Mater. 2020, 32, 2003879. 10.1002/adma.202003879 33206429

[143] Q. Liu , W. Dengv , Y. Pan , C. Sun , Chem. Sci. 2020, 11, 6045. 34094097 10.1039/d0sc01474e PMC8159323

[144] S. Lee , J. Lee , J. Kim , M. Agostini , S. Xiong , A. Matic , J. Y. Hwang , Energies 2020, 13, 2791.

[145] N. Xiao , X. Ren , W. D. McCulloch , G. Gourdin , Y. Wu , Acc. Chem. Res. 2018, 51, 2335. 30178665 10.1021/acs.accounts.8b00332

[146] X. Ren , Y. Wu , J. Am. Chem. Soc. 2013, 135, 2923. 23402300 10.1021/ja312059q

[147] X. Ren , K. C. Lau , M. Yu , X. Bi , E. Kreidler , L. A. Curtiss , Y. Wu , ACS Appl. Mater. Interfaces 2014, 6, 19299. 25295518 10.1021/am505351s

[148] X. Ren , M. He , N. Xiao , W. D. McCulloch , Y. Wu , Adv. Energy Mater. 2017, 7, 1601080.

[149] N. Xiao , G. Gourdin , Y. Wu , Angew. Chem. Int. Ed. 2018, 57, 10864. 10.1002/anie.201804115 29787628

[150] W. Wang , N. C. Lai , Z. Liang , Y. Wang , Y. C. Lu , Angew. Chem. Int. Ed. 2018, 130, 5136.

[151] J. Stevens , B.‐E. Mellander , Solid State Ionics 1986, 21, 203.

[152] A. Chandra , Indian J. Phys. 2016, 90, 759.

[153] P. Kesharwani , D. K. Sahu , M. Sahu , T. b. Sahu , R. Agrawal , Ionics 2017, 23, 2823.

[154] H. Fei , Y. Liu , Y. An , X. Xu , G. Zeng , Y. Tian , L. Ci , B. Xi , S. Xiong , J. Feng , J. Power Sources 2018, 399, 294.

[155] H. Fei , Y. Liu , Y. An , X. Xu , J. Zhang , B. Xi , S. Xiong , J. Feng , J. Power Sources 2019, 433, 226697.

[156] Y. Pavani , M. Ravi , S. Bhavani , R. Karthikeya , V. N. Rao , J. Mater. Sci. Mater. Electron. 2018, 29, 5518.

[157] M. Rayung , M. M. Aung , A. Ahmad , M. S. Su'ait , L. C. Abdullah , S. N. A. M. Jamil , Mater. Chem. Phys. 2019, 222, 110.

[158] K. V. Kumar , G. S. Sundari , J. Eng. Sci. Technol. 2010, 5, 130.

[159] A. Chandra , Polym. Bull. 2017, 74, 4815.

[160] R. Singh , J. Baghel , S. Shukla , B. Bhattacharya , H. W. Rhee , P. K. Singh , Phase Transitions 2014, 87, 1237.

[161] M. Ravi , S. Bhavani , K. K. Kumar , V. N. Rao , Solid State Sci. 2013, 19, 85.

[162] N. Reddeppa , A. Sharma , V. N. Rao , W. Chen , MEAS 2014, 47, 33.

[163] R. Nadimicherla , R. Kalla , R. Muchakayala , X. Guo , Solid State Ionics 2015, 278, 260.

[164] S. S. Basha , G. S. Sundari , K. V. Kumar , Mater. Today Proc. 2016, 3, 11.

[165] A. Chandra , A. Chandra , A. Bhatt , S. Basak , M. Khan , Mater. Today Proc. 2020, 33, 5046.

[166] Y. Pavani , M. Ravi , S. Bhavani , A. Sharma , V. V. R. N. Rao , Polym. Eng. Sci. 2012, 52, 1685.

[167] J. M. Doux , L. Leguay , A. L. G. La Salle , O. Joubert , E. Quarez , Solid State Ionics 2018, 324, 260.

[168] T. Masese , K. Yoshii , Y. Yamaguchi , T. Okumura , Z.‐D. Huang , M. Kato , K. Kubota , J. Furutani , Y. Orikasa , H. Senoh , Nat. Commun. 2018, 9, 3823. 30237549 10.1038/s41467-018-06343-6 PMC6147795

[169] Q. Zhao , S. Stalin , C.‐Z. Zhao , L. A. Archer , Nat. Rev. Mater. 2020, 5, 229.

[170] T. Oshima , M. Kajita , A. Okuno , Int. J. Appl. Ceram. Technol. 2004, 1, 269.

[171] Y. Y. Yao , J. Kummer , J. Inorg. Nucl. Chem. 1967, 29, 2453.

[172] X. Lu , M. E. Bowden , V. L. Sprenkle , J. Liu , Adv. Mater. 2015, 27, 5915. 26305734 10.1002/adma.201502343

[173] J.‐M. Doux , L. Leguay , A. L. G. La Salle , O. Joubert , E. Quarez , Solid State Ionics 2018, 324, 260.

[174] C. Delmas , C. Fouassier , J. M. Reau , P. Hagenmuller , Mater. Res. Bull. 1976, 11, 1081.

[175] H. Yuan , H. Li , T. Zhang , G. Li , T. He , F. Du , S. Feng , J. Mater. Chem. A 2018, 6, 8413.

[176] J. B. Goodenough , H. P. Hong , J. Kafalas , Mater. Res. Bull. 1976, 11, 203.

[177] U. Guth , B. Löscher , P. Schmidt , H. Wulff , H.‐H. Möbius , Solid State Ionics 1992, 51, 183.

[178] M. Avdeev , M. Sale , S. Adams , R. P. Rao , Solid State Ionics 2012, 225, 43.

[179] R. Eremin , N. Kabanova , Y. A. Morkhova , A. Golov , V. Blatov , Solid State Ionics 2018, 326, 188.

[180] R. Xiao , H. Li , L. Chen , J. Mater. Chem. A 2020, 8, 5157.

